# Detecting co-selection through excess linkage disequilibrium in bacterial genomes

**DOI:** 10.1093/nargab/lqae061

**Published:** 2024-06-06

**Authors:** Sudaraka Mallawaarachchi, Gerry Tonkin-Hill, Anna K Pöntinen, Jessica K Calland, Rebecca A Gladstone, Sergio Arredondo-Alonso, Neil MacAlasdair, Harry A Thorpe, Janetta Top, Samuel K Sheppard, David Balding, Nicholas J Croucher, Jukka Corander

**Affiliations:** Department of Biostatistics, University of Oslo, Oslo, Norway; Department of Biostatistics, University of Oslo, Oslo, Norway; Department of Biostatistics, University of Oslo, Oslo, Norway; Norwegian National Advisory Unit on Detection of Antimicrobial Resistance, Department of Microbiology and Infection Control, University Hospital of North Norway, Tromsø, Norway; Oslo Centre for Biostatistics and Epidemiology, Oslo University Hospital, Oslo, Norway; Department of Biostatistics, University of Oslo, Oslo, Norway; Department of Biostatistics, University of Oslo, Oslo, Norway; Department of Biostatistics, University of Oslo, Oslo, Norway; Department of Biostatistics, University of Oslo, Oslo, Norway; Department of Medical Microbiology, UMC Utrecht, Utrecht, The Netherlands; Ineos Oxford Institute of Antimicrobial Research, Department of Biology, University of Oxford, Oxford, United Kingdom; Melbourne Integrative Genomics, School of BioSciences and School of Mathematics & Statistics, University of Melbourne, Parkville, Victoria, Australia; Department of Infectious Disease Epidemiology, School of Public Health, Imperial College London, United Kingdom; MRC Centre for Global Infectious Disease Analysis, School of Public Health, Imperial College London, United Kingdom; Department of Biostatistics, University of Oslo, Oslo, Norway; Parasites and Microbes, Wellcome Sanger Institute, Cambridge, UK; Helsinki Institute of Information Technology, Department of Mathematics and Statistics, University of Helsinki, Helsinki, Finland

## Abstract

Population genomics has revolutionized our ability to study bacterial evolution by enabling data-driven discovery of the genetic architecture of trait variation. Genome-wide association studies (GWAS) have more recently become accompanied by genome-wide epistasis and co-selection (GWES) analysis, which offers a phenotype-free approach to generating hypotheses about selective processes that simultaneously impact multiple loci across the genome. However, existing GWES methods only consider associations between distant pairs of loci within the genome due to the strong impact of linkage-disequilibrium (LD) over short distances. Based on the general functional organisation of genomes it is nevertheless expected that majority of co-selection and epistasis will act within relatively short genomic proximity, on co-variation occurring within genes and their promoter regions, and within operons. Here, we introduce LDWeaver, which enables an exhaustive GWES across both short- and long-range LD, to disentangle likely neutral co-variation from selection. We demonstrate the ability of LDWeaver to efficiently generate hypotheses about co-selection using large genomic surveys of multiple major human bacterial pathogen species and validate several findings using functional annotation and phenotypic measurements. Our approach will facilitate the study of bacterial evolution in the light of rapidly expanding population genomic data.

## Introduction

The rapid rate of evolution of bacterial genomes has made them a popular target of studying selection in both experimental and natural populations. The emergence of affordable high-resolution population genomics a decade ago ushered us into a new era with improved possibilities to investigate both microscopic and macroscopic evolution of bacterial genomes, including for example codon bias (1), intergenic selection (2) and variation in gene content (3). Despite steady progress in genome-wide association study (GWAS) methodologies specifically designed for bacterial populations (4,5), the difficulty of measuring quantitative phenotypic variation in large numbers of isolates has restricted the use of GWAS mostly to the study of antibiotic resistance, with a few exceptions covering for example duration of colonization (6), genome-wide transcriptomics (7) and virulence (8). Traits such as reproductive rate, survival and transmissibility are of key interest in bacteria but remain difficult to measure in sufficient numbers in natural populations. Motivated by this obstacle, phenotype-free approaches to uncovering signals of selection have been introduced (9–12), based on the rationale that positively selected variation in complex traits would likely be caused by synchronized changes in multiple genes and/or regulatory elements that are detectable from excess linkage disequilibrium (LD) between distant sites across a sample of genomes. This provides a methodological toolkit complementary to GWAS enabling analyses termed genome-wide epistasis and co-selection studies (GWES), which has been recently used to unravel signals of selection due to epistasis for a wide diversity of bacteria (13–16). Arnold *et al.*, using positive, negative and sign epistasis models, demonstrated that under selection, even relatively weak epistasis is sufficient for driving adaptation in moderately to highly recombinogenic bacteria, which provides a more theoretical justification for GWES analysis (17), see also the recent work on the possibly saltational role of epistasis in highly recombining bacterial species (18).

Existing GWES approaches are limited to discovering links between distant loci within the region where LD asymptotes towards its lower bound. However, many co-evolving loci are organized into clusters of genes (e.g. co-transcribed in operons). Therefore, studying only long-range links ignores most of the allelic co-variation occurring in genomes. A fine-scale haplotype structure analysis of *Neisseria gonorrhoeae* indeed revealed numerous likely examples of positive co-selection in different regions of the chromosome of this highly recombinant species, and extensive population genetic simulations suggested that such LD patterns could be explained by either directional selection on horizontally acquired alleles, or balancing selection maintaining the diversity (19). Motivated by these insights, we aimed at developing a scalable statistical approach that disentangles neutral LD from co-selection and epistasis at any genomic distance. Apart from co-selection and epistasis, LD can also be influenced by various other factors, including population structure, population expansion, mutation and admixture. While disentangling neutral LD remains a valuable approach for identifying co-selection and epistasis, sole reliance on it cannot distinguish between underlying evolutionary factors. Since wet-lab-based validation would typically be necessary to resolve causal factors underlying observed deviations from a baseline neutral LD, this serves as a useful starting point towards identifying important molecular drivers of success in bacterial populations.

GWES methods generally exploit the decay of LD as a function of genomic distance to label SNP pairs as ‘outliers’ with respect to the background distribution of LD estimated from population data. The intuition here, for example in the context of synergistic epistasis, is that when the combined effect on selection of two or more polymorphic loci is greater than the sum of their individual effects, this allele combination will be maintained in association in the population, giving rise to discernable patterns of LD. Similar to Arnold *et al.* (19), it is possible to extend the notion of ‘outlier’ LD level to SNPs in close proximity to each other by simulating the distribution of LD strength as a function of base pair distance using a neutral Wright-Fisher model. These simulations approximate the population level co-variation of alleles expected under neutrality and can be used to screen pairs of loci for outliers that may be due to co-selection. We show that this approach works well and maintains a low false positive rate. However, as fitting of the neutral model parameters and forward simulation of the fitted model in a sufficiently large number of replicates is computationally costly, we developed an empirical model-free approximation that is scalable to large population genomic datasets. The model-free method is motivated by the common assumption that a majority of the observed LD within a bacterial population reflects near-neutrality (20–23), which implies that a majority of observations are not strongly influenced by selection. As a result, it is possible to analyse and interpret LD patterns without relying on additional assumptions about underlying genetic models or selection pressures. By extension, it then becomes feasible to use the empirical LD decay distribution to call outliers. Moreover, the approach accounts for heterogeneity in evolutionary rates, such as mutation and recombination hotspots.

The model-free algorithm is implemented as an open-source R package ‘LDweaver’ (https://github.com/Sudaraka88/LDWeaver), which can be used to perform a comprehensive GWES in large-scale bacterial datasets. LDWeaver incorporates the functionality of the popular GWES package Spydrpick for long-range LD outlier detection (12) and extends this by allowing analysis of LD at any genomic distance. LDWeaver provides automated functional annotations on all putative co-selected SNPs and generates an array of visualizations to allow users to efficiently explore the results. We use published population genomic data for the major human pathogens *Streptococcus pneumoniae* (24,25), *Campylobacter jejuni* (26), *Escherichia coli* (27) and *Enterococcus faecalis* (28) to identify both known and novel signals of co-selection linked to the molecular basis of pathogenicity, survival and other key bacterial phenotypes.

## Materials and methods

### Measuring LD

By default, LDWeaver removes sites with MAF <0.01 and gap frequency >0.15. Sites with ‘gap’ as the second most common allele are also discarded from the analysis by default, but LDWeaver has a filtering option called ‘relaxed’ that retains these sites in the analysis. This option could be particularly useful for alignments with a limited number of SNPs, as gaps can reflect insertions or deletions with functional effects (80).

Following SpydrPick (12), we use MI to measure LD. The pairwise MI between two sites (modelled as discrete random variables) is given by:


(1) \begin{eqnarray*}{\mathrm{MI}}\left( {X,Y} \right) = \mathop \sum \limits_{x \in X} \mathop \sum \limits_{y \in Y} p\left( {x,y} \right)log\ \left( {\frac{{p\left( {x,y} \right)}}{{p\left( x \right)p\left( y \right)}}} \right)\end{eqnarray*}

Where $X$ and $Y$ denote two sites with alleles $x \in X$ and $y \in Y,$ respectively. For SNP data, the alphabet comprises nucleotides A, C, G, T and the gap character N. Here $p( {x,y} )$denotes the joint probability of $x = X$ and $y = Y$ and $p( x ),p( y )$ are the corresponding marginal probabilities which are estimated from count data (12).

Let ${n}_s$ denote the number of sequences in the alignment, then:


(2) \begin{eqnarray*}\hat{p}\left( {x,y} \right) = \frac{{n\left( {x,y} \right) + 0.5}}{{{n}_s + {r}_x{r}_Y \times 0.5}}\end{eqnarray*}

where $n( {x,y} ) = | {{S}_{xy}} |$ is the number of sequences with $X = x$ and $Y = y$, ${r}_X = | X |$ and ${r}_Y = | Y |$.

Strong population structure in bacteria presents a problem for analysis (80). We adopt a widely-used sequence-reweighting approach (10–12,81,82). The weight ${w}_i \in [ {1/n,1} ]$ for sequence $i$ is computed as the reciprocal of the number of sequences with mean per-site Hamming distance $ <\, t$, where $t$ is a dataset-dependent threshold that typically satisfies $t \in ( {0.1,0.25} )$. This population structure correction is applied to the LD structure estimation by substituting effective counts in Eq. (2) given by ${n}_s = \mathop \sum \limits_{i = 1}^n {w}_i$ and $n( {x,y} ) = \mathop \sum \limits_{i \in {S}_{xy}}^{} {w}_i$.

In practice, the MI computation process is optimised using a sparse matrix representation and performed in blocks of SNPs (83), which can be feasible even in systems with relatively low memory. LDWeaver requires genome-wide short-range links to be retained in memory (see below), but most long-range links with low MI values are discarded after processing each SNP block. The user specifies an approximate number of long-range links to be retained for downstream analysis.

### Modelling short-range links.

By default, SpydrPick (12) uses $S$ = 10 kb as the threshold for defining a short-range link, but in LDWeaver we chose $S = 20{\mathrm{\ }}$ kb as the default threshold to better capture the region of rapid LD decay. The user can adjust this parameter as required.

**Figure 1.**
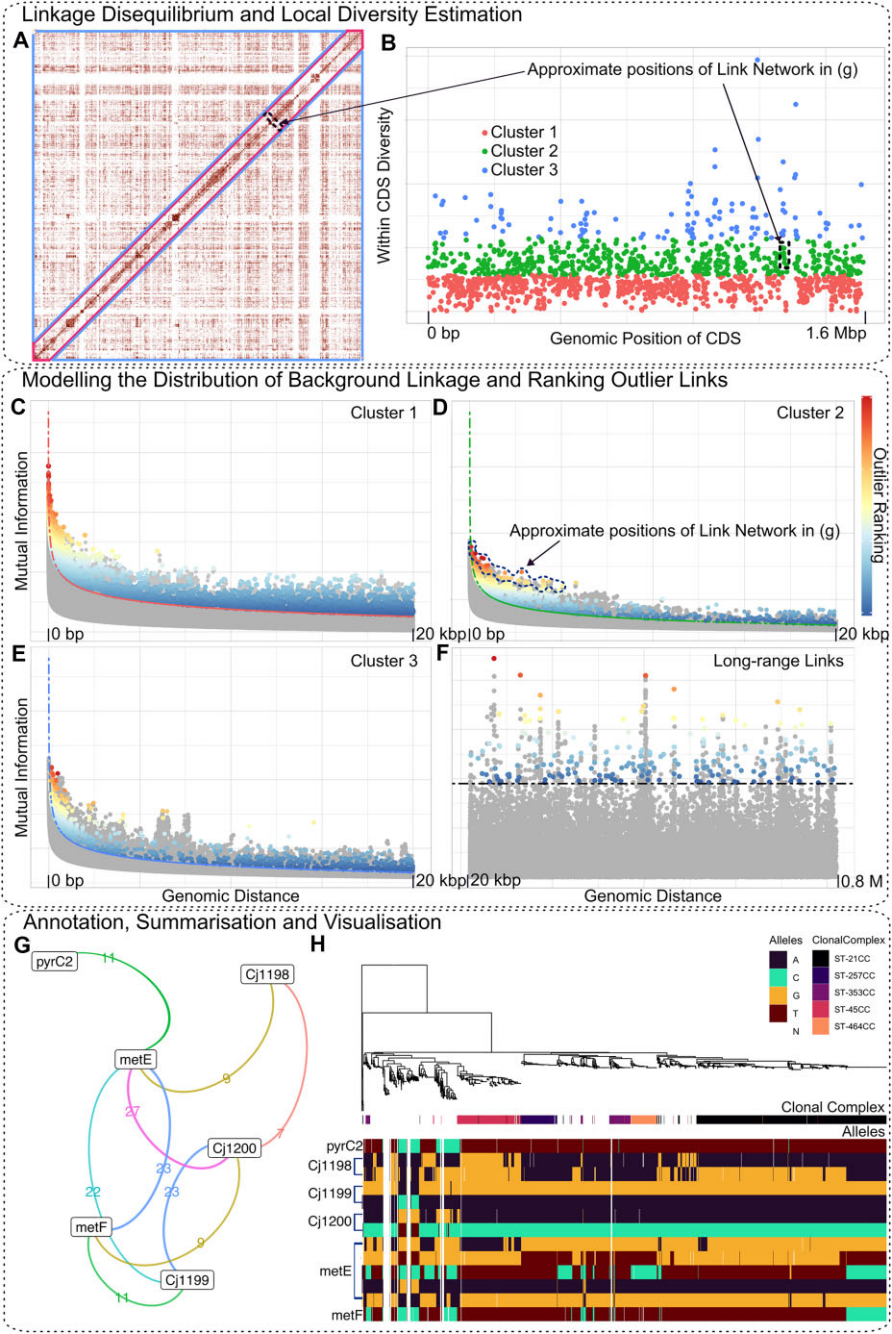
Overview of the LDWeaver pipeline. (**A**) Genome-wide linkage disequilibrium (LD) for 1480 *Campylobacter jejuni* isolates (26). LD is measured using mutual information (MI) and a weighting strategy is employed to account for population structure. The axes correspond to genomic positions in the NCTC 11168 reference genome, and the colour intensity reflects the strength of LD. Blue triangles outline regions of long-range LD, while the short-range high-LD region is outlined in red. (**B**) Genomic diversity (measured using Hamming distance compared to the reference) within each coding region (CDS) is used to account for local variation in short-range background LD. Each point corresponds to a CDS, and the vertical axis shows the average number of sites that differ from the reference. K-means clustering is used to divide the CDS into three clusters (see legend). Sites from intergenic regions are allocated to the nearest cluster. (**C–** **F**) GWES Manhattan plots show the distribution of LD measured using weighted MI (*y*-axis) at varying genomic distances (*x*-axis). For better visualisation in this overview figure, numeric values are removed from the *y*-axis. All links (between synonymous, non-synonymous and intergenic sites) are included in these plots. Plots are shown for short-range links in the three clusters (C–E) and long-range links (F). Modelled background LD is shown respectively using red, green, blue, and black dashed lines, respectively. The colour shading of each point indicates the ranking given to outlier links (see the colour bar in the rightmost panel - numeric values removed to reduce clutter). The topmost (bright red) colour signifies rank 1, highlighting the most extreme outlier. Decreasing ranks follow the colour bar from top-bottom. For short- and long- range analyses, link ranking is based on the estimated short-range *P*-value and the measured MI, respectively (see Materials and methods). Links that are either inferred as indirect or with MI below the background LD are shown in grey and not ranked. In (F) the background LD is invariant to genomic distance and computed using the Tukey criteria (dashed black line). (**G**) The LDWeaver network plot generated for *metE* summarises all the outlier links involving a site in the gene. Here, the edges are coloured based on the number of links between linked genomic region nodes (see *Campylobacter* results section). (**H**) Investigating the allele distribution within the linked region (alleles panel) shows that several deletions and mutations observed within several clonal complexes are driving the high LD signal picked up by LDWeaver. This and the subsequent phylogenetic trees shown in this manuscript were generated using FastTree 2 (102).

Additionally, LDWeaver accounts for genome-wide variation in local LD patterns, which can arise due to varying mutation and/or recombination rates, by introducing a clustering and segregation step. First, for each coding sequence (CDS) segment in the annotations file, the per-site number of mismatches between the CDS and its reference sequence (i.e. the per-site Hamming distance) is computed. Next, the CDS are clustered using the *k*-means algorithm (84). Due to the challenges in accounting for heterogeneity stemming from population structure (80), LDWeaver avoids estimating this parameter and the choice of the number of clusters is a user modifiable (default $k = 3$). A user can avoid clustering by setting the $k = 1$. Generally, the LDWeaver output with CDS diversity and clustering (similar to Figure 1B) can be useful to determine *k*. In Supplementary Figures S2, S3, S6, S8 and S9, for each dataset analysed, we show our choice of *k* and the output CDS diversity and clustering plot. Generally, increasing $k$ beyond a sensible value will have a limited impact on final analysis, provided that enough CDS remain in all clusters to approximately estimate the decay of background LD.

Since CDS regions exclude intergenic regions, each intergenic SNP is manually merged into the cluster closest to its genomic location.

We used the *C. jejuni* dataset to compare the modelling of short-range rapid LD decay between (1) the LDWeaver model-free approach and (2) computationally demanding neutral simulations. For each cluster, we used mcorr (85) to estimate the parameter triplet: mutation rate, recombination rate and the recombination tract length. For each parameter triplet, 100 neutral replicates were generated using bacmeta (86). In each simulation 20 subpopulations of bacteria, each comprising 1000 individuals with a 200 kb gen-me were included to generate a sufficiently large and diverse alignment. Migration rate probability was set at the default 0.01. Each simulation was performed for 20 000 generations to ensure convergence and then 1000 individuals were sampled. The mean LD decay was directly estimated from this neutral data as a function of bp-sep (base-pair separation).

LDWeaver directly models the LD-decay using the genomic alignment, separately for each cluster. At each discrete bp-sep, the 95th percentile (*q*_95_) MI value is extracted from the genomic data. Here, *q*_95_ was chosen intuitively as a reasonable choice to model the background LD (87). Next, the linear model: ${\mathrm{log}}\ ( {{q}_{95}} )\ \sim {\mathrm{log\ ( {bp\_sep}}} )$ is fitted and the exponent of the fitted values of this model (i.e.${\hat{q}}_{95}$) is chosen as the bp-sep dependent short-range background-LD threshold. The 95th percentile is chosen to be modelled because it is close to the upper tail of the distribution, which is the region of interest, but little affected by large outliers so that the fitted curve is reasonably smooth.

### Outlier calling and link ranking.

Outlier calling is performed at each discrete bp-sep value. Let $d \in [ {1,S} ]$ denote the bp-sep of interest, let $L( d )$ denote all the links that are $d$ bp-sep apart and let ${L}^*( d )$ denote the subset with ${\mathrm{MI}}( {L( d )} ) \ge {\hat{q}}_{95}( d )$. First, the model: ${\mathrm{MI}}( {L( d )} )\ - \ {\hat{q}}_{95}( d )\sim Beta( {\alpha ,\beta } )$ is fitted (88) in order to compute an approximate, $\forall {L}^*( d ),$ a short-range p-value (referred to as ‘srp’ in LDWeaver to denote short-range p-value). Links with ${\mathrm{MI}} <\, {\hat{q}}_{95}( d )$ are always discarded and the user can choose a srp cut-off value (default $p = 1e - 3$) to further reduce the set of links retained.

Although a model-free, permutation analysis is available to compute the srp, it would greatly add to the computational burden for large bacterial genomic datasets and is not required because the Beta distribution provides a good approximation. We confirmed this empirically by performing multiple trials comparing permutation-based and beta-approximation p-values using the descdist() function available in the R package, fitdistrplus (88).

The ${\hat{q}}_{95}$ values are modelled separately for each genome cluster. The srp for links between sites from the same cluster is computed using the LD-decay model fitted to data from that cluster. When a link spans two clusters, the maximum of the two srp values is used.

### Filtering indirect links

Because of its success in inferring gene expression networks (89) and its utility in SpydrPick, we added ARACNE as a filtering step to the LDWeaver pipeline to overcome the inability of pairwise methods to distinguish ‘direct’ associations. For a dataset with 100 000 SNPs, MI values will be computed for approximately 5 billion unique links. When performing GWES, the interest is typically focussed on links with high MI values (i.e. with larger than normal linkage). However, many of these links will be driven by the same underlying association. Identifying the causal link is extremely challenging, especially for bacterial data due to factors such as clonality and genome plasticity (80).

Given multiple links that can be explained by the same underlying causal effect, we use ARACNE to retain only the link with the strongest signal (called a ‘direct’ link). This can reduce the number of links retained by several orders of magnitude, greatly reducing the manual curation task. ARACNE scans the entire LD landscape and a link $( {X,Y} )$ is considered to be indirect if ${\mathrm{MI}}( {X,Y} ) \le [ {{\mathrm{MI}}( {X,Z} ),{\mathrm{MI}}( {Z,Y} )} ]$ for any SNP Z. A detailed explanation of the ARACNE algorithm and this specific filtering step is available in (12).

### Detecting outliers in long-range links

Long-range (bp-sep >$S$) background LD varies little with bp-sep, so the genome clustering step that was used to model short-range links is not required. To determine a background-LD threshold (12), we adopt an approach similar to that of SpydrPick in which LDWeaver computes the Tukey (90) outlier threshold: ${T}_1 = {Q}_1 + 1.5 \times IQR$, where $IQR = {Q}_1 - {Q}_3$ and ${Q}_1$ and ${Q}_3$ are the first and third quartiles, respectively. Links with $MI >{T}_1$ are directly ranked based on the MI value. In cases where < 5000 links surpass ${T}_1$, LDWeaver retains the top 5000 (default) links based on MI value.

The Tukey outlier detection approach is simple, but we prefer it to a permutation approach for two reasons. Firstly, this threshold has limited impact on the long-range GWES analysis itself. While an outlier is defined based on whether its MI value passes this threshold, it has no bearing on link ranking itself. Downstream analysis and the eventual manual curation can be performed on the highest-ranked subset of ARACNE direct links without requiring a threshold. Secondly, due to the high degree of LD observed in bacteria, the background MI values computed from the observed MSA could be larger than the null MI values computed from a label-shuffled model. Therefore, the computed permutation threshold could potentially be too conservative.

While LDWeaver can perform GWES analysis on both short- and long-range links, it is fully compatible with the SpydrPick output for long-range link analysis. For a dataset that has already been analysed using SpydrPick, users have the option to first perform only the short-range analysis in LDWeaver, then present the SpydrPick output as input to LDWeaver to perform the downstream analyses of long-range links.

### Downstream analyses and visualizing putative links and sites.

We have integrated several powerful R visualization tools into LDWeaver (91,92). To prioritize and perform wet-lab based modelling and validation, it is helpful to understand the functional annotations of the SNPs involved in high-MI links. Therefore, functional annotations are added to all such SNPs using SnpEff (29). Additionally, LDWeaver classifies each SNP as non-synonymous, synonymous, or intergenic. The non-synonymous versus synonymous distinction is based on the most common allele at multi-allelic sites. Based on this classification, LDWeaver generates an additional output comprising, by default, a set of 250 top ranked links excluding links between two synonymous sites.

GWES Manhattan plots were introduced to visualize the distribution of MI as a function of bp-sep (12). LDWeaver generates the long-range GWES plot introduced in SpydrPick, along with two plots for short-range links. The first short-range plot is similar to the long-range GWES plot but with points shaded according to the srp value. The second plot shows the segregation of links into genomic clusters. While these plots are less informative compared to Manhattan plots in genome-wide association studies (GWAS) due to the lack of genomic positions, they can be useful to assess the LD-decay fit between ${\hat{q}}_{95}$and the genomic data.

LDWeaver also generates a linear tanglegram using the R package ChromoMap (93) to indicate the genomic positions of top ranked links in the short range. These tanglegrams span the whole genome and are broken down into segments for improved utility. Additionally, LDWeaver generates a figure depicting a genome-wide overview of the LD structure in the dataset. First, a sparse, SNP-level LD matrix is created using the MI values of the saved link data. Then, an averaging kernel is applied to reduce the dimensions of the matrix to approximately $1000 \times 1000$ (94). The resulting matrix is plotted in the form of a heatmap with SNP positions as x and y labels (95). This bird's-eye view can be useful in some analyses to quickly identify high-LD regions and blocks.

Additionally, LDWeaver generates a network plot for top ranked links using the R package ggraph (96). The gene-level regions of each site are extracted from the annotations file and the links between these regions are coerced into a network (97). To reduce clutter in the network plot, edges with only 1 link between nodes are removed. Afterwards, any nodes with no edges between them are also dropped from the plot. The edges are coloured to depict the number of links between genes. Furthermore, the edge width and transparency are also moderated to reflect the MI value of the highest ranked link between the two regions. Furthermore, LDWeaver provides the option to generate a similar gene network for any chosen gene. The user can decide whether to use short-range, long-range or both link lists to generate this plot.

Using a user-provided phylogeny, LDWeaver can generate a tree-plot of user-determined putative sites and phenotypes. This type of plot is inspired by some of the visualization options available in Microreact (98) and has also been widely used in the previous GWES literature (11,15). The phylogeny will be midpoint rooted by default (99,100) and the SNP data, user provided phenotypes are sorted in the same order as the phylogeny. Finally, the plot is generated using the R package ggtree (101).

Finally, LDWeaver generates the output required to dynamically visualize links using the R package GWESExplorer (https://github.com/jurikuronen/GWES-Explorer). This Node.js based shiny app can be used to generate the GWES Manhattan plot, circular tanglegram and the tree-plot for an arbitrarily chosen subset of putative links.

### Runtime

A complete LDWeaver analysis with default parameters on a dataset comprising 2000 sequences with 80 000 SNPs on average requires 4756 s (∼80 min) on a computer with 32GB of ram and 10 parallel CPU cores running R version 4.2.2 with openBLAS v0.3.21 support.

## Results

### Overview of LDWeaver

Performing GWES analysis using LDWeaver requires two inputs, a multiple sequence alignment (MSA) and the annotation file of the reference in Genbank or Gff3 format. Initially, LDWeaver filters out sites with low minor allele frequency (default: 0.01) and high gap frequency (default: 0.15), with an option for a ‘relaxed’ filter to retain sites with gap (N) as the second most common allele. Following SpydrPick (12), LD between each SNP pair is measured using mutual information (MI). To address population structure, a sequence-reweighting approach is applied, where the weight for each sequence is computed as the reciprocal of the number of sequences with a mean per-site Hamming distance below a user definable threshold (default: 0.1).

Generally, SNPs in genomic proximity tend to have very high LD, but LD levels rapidly decline with base-pair distance to a constant value for all long-range SNP pairs (see Figure 1A). First, LDWeaver uses a user-definable genomic distance threshold to classify short range links (default: links between sites < 20 kb apart are considered short range). To determine a threshold for outlier calling, it is necessary to model the decay in LD with genomic distance and the shape of this decay is determined by many factors. These may include the type of species, population structure, local mutation and recombination rates, variation in gene content and selection pressures. To account for this heterogeneity, LDWeaver measures the per-site mean Hamming distance within coding regions around the chromosome and clusters them using *k*-means based on the estimated local diversity (see Figure 1B). Based on the output in Figure 1B (generated by LDWeaver), the user has the option to select the most appropriate number of clusters for the dataset (default: 3).

Background decay in LD is modelled separately for each cluster. Here, a linear model is fitted to log transformed 95th percentile MI values and the corresponding base pair separation. Afterwards, the exponent of fitted values is used as the base pair separation dependent background LD (see Figure 1C–E). Since long-range background LD is uniform (see Figure 1F), the Tukey outlier approach in SpydrPick can be used to estimate the background LD.

After calling outliers based on the estimated background LD, LDWeaver provides a list of locus pairs ranked in order of strength of evidence for co-selection. This ranking is based on the outlier short-range *P*-value (see Materials and Methods). LDWeaver includes several options to ease the task of generating a list of potential epistatic SNP-pairs for expert manual curation and wet lab validation. First, all outlier links are annotated using SnpEff (29) and links that include a non-synonymous substitution are given a higher priority. Afterwards, this SNP classification is leveraged to generate an additional output comprising a set of (default: 250) top-ranked links after discarding links between two synonymous sites. All short-range results presented in this manuscript using real data are based on analysing these top 250 most significant links. Furthermore, LDWeaver summarizes links into networks (see Figure 1G), which helps to prioritize the most promising genome regions. Finally, LDWeaver can be used to visualize the allele distributions within these networks (see Figure 1H).

### LDWeaver detects co-selection signals in simulated genomes.

With the detection of long-range links validated previously (12), the primary objective here is to detect links short-range under epistasis by looking for signs of co-selection between SNPs. We validated LDWeaver using Wright-Fisher models representing several evolutionary scenarios that were simulated using SLiM version 4 (30). Although the original design of SLiM does not support simulating bacterial evolution, recent advances (31) now enable considering circular genomes, horizontal gene transfer, and bacterial recombination. Notably, SLiM is one of the only available options with the ability to simulate epistasis in bacterial populations and generate full genome alignments as output, which is necessary for LDWeaver analysis.

To make the simulations as realistic as possible, we chose the first 200 kb from the ATCC 700669 (*S. pneumoniae*) reference genome as the ancestral sequence. Each simulation comprised 10 000 isolates, equally distributed across 10 subpopulations. Recombination tract lengths were drawn from a geometric distribution with a mean of 500 bp, while mutation and recombination rates were fixed at 2e-7 (per bp, per generation) and 8e-7 (per bp, per generation), respectively. The simulations allowed migration between all 10 subpopulations at a rate of 5e-2 (per bp, per generation).

Introducing positive or negative *synergistic* epistasis alone led to a loss of genetic diversity through fixations after several generations, which stands in contrast with observations in real bacterial populations (32). While such epistatic interactions are undoubtedly present in bacterial populations, these simulations lack the complexity needed to model the processes that continuously shape the LD within populations. To address this and to maintain more realistic levels of genetic diversity, we opted to simulate a balancing selection scenario employing a version of negative multiplicative (synergistic) epistasis.

**Figure 2.**
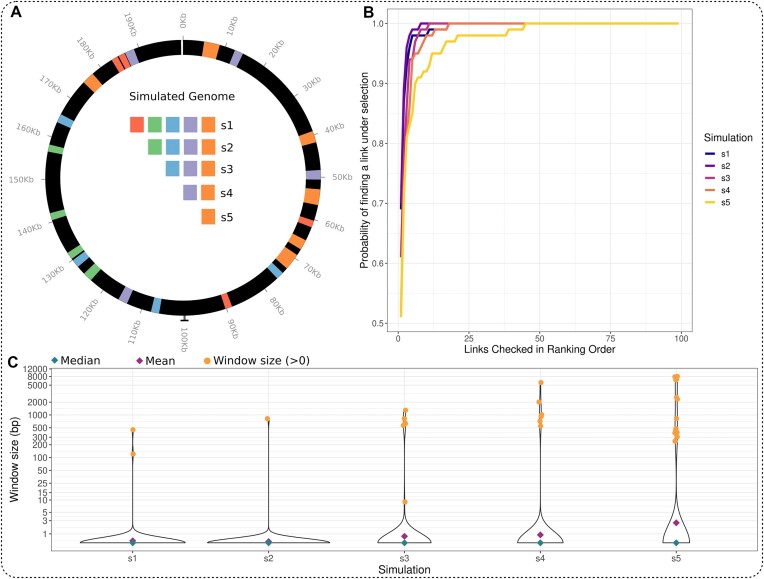
Validation of LDWeaver using bacterial population simulations. (**A**) Simulated genomic region (first 200kb of ATCC700669) showing the target Coding Sequences (CDS). In each simulation scenario, grouped target CDS (depicted by colours) were chosen for epistatic interactions. In **s1**, all 22 CDS selected and in **s5**, only the 6 CDS in the orange group were selected (see main text for a detailed breakdown). (**B**) For each simulation (see legend), each curve shows the variation between the number of links checked in ranking order (*x*-axis) and the probability of detecting a ‘target link’, a link from within or between two target regions (*y*-axis). This reaches 1 when all replicates contain a target link. All replicates from **s1–** **s4** and 97% of replicates from the most challenging **s5** contain a target link within the *top 20 ranked*. The challenge in **s5** is 2-fold: only < 10% of the genome is under epistasis, and only two orange regions near position 70 Kb (panel a) can contribute to ‘between target-region’ short-range links. (**C**) For each simulation scenario (*x*-axis), *y*-axis shows the genomic distance between sites in the top 5 ranked links and the closest target region. Most replicates in **s1–** **s5** contain at least one target link in the *top 5 ranked* (blue diamond shows median = 0, purple diamond shows mean < 3, which increases from **s1** to **s5**). Each orange dot corresponds to a replicate that does not contain a target link, and the *y*-axis shows the minimum genomic distance from a target region to a site. To elaborate, two replicates in s1 do not contain a target region link in the *top 5 ranked*, and the closest site in each case is approx. 100 and 500 bp to a target region.

We identified the 22 longest coding regions (CDS) from within the first 200kb of the ATCC700669 reference genome, each with length >1500 bp, as *potential* ‘target regions’ for epistatic interactions. The distribution of these 22 regions on the genome is illustrated in Figure 2A. Initially, each CDS was randomly allocated to one of five groups represented here using different colours: magenta, green, blue, purple and orange (see Figure 2). Utilizing these groups, we constructed five simulation scenarios, denoted as **s1**-**s5**. In **s1**, all 22 CDS from the five groups were selected as *targets*, covering a combined target region length of 23.8% of the total genome. For **s2**, four groups were chosen, resulting in 18 target CDS covering 20.8% of the genome. Similarly, **s3** involved three groups with 14 CDS with 17.5% coverage, **s4** involved two groups with 10 CDS with 13.7% coverage, and **s5** involved only one group with six CDS and 9.5% coverage. This design ensures that **s5** is considerably more challenging than **s1** because only two orange regions near 70 Kb (see Figure 2A) can contribute to ‘between target-region’ short-range links. The remaining target links in **s5** are from ‘within target-regions’.

For all simulation scenarios, three types of mutations were introduced to the population. All regions *outside* the target CDS were assigned mutation type **m1**, which had a *slightly* deleterious selection coefficient of −0.00001, reflecting the assumed near-neutrality in bacterial genomes. The target CDS were randomly allocated either mutation type **m2** or **m3**, both were beneficial with an additive selection coefficient of 0.001. Since both mutation types had the same effect, in the absence of epistasis, they would fix after several generations. An epistatic interaction was introduced between **m2** and **m3**; carrying both will result in a 5% reduction in fitness.

The simulation was continued for 20000 generations and 1000 genotypes were sampled at the end. Each scenario (**s1–** **s5**) was replicated 100 times, totalling 500 simulations. At the end of each replicate, the extracted genome alignment was used for LDWeaver analysis. Importantly, since this simulation does not account for the complexities of amino acid modifications, SnpEff annotations were avoided, and no distinctions were made between synonymous and non-synonymous mutations.

In **s1**, only 2.4% of examined links were true target links (i.e. a link from within or between two target CDS regions), which reduced to 1.3% for **s5** (see Supplementary Table S1). Since LDWeaver is primarily a link ranking algorithm, its performance was evaluated based on its ability to include target links in the top subset of ranked links. This best aligns with the analysis method used for real biological data presented in the manuscript.

For scenarios **s1–** **s5**, 70.4%, 62.6%, 61.8%, 52.4% and 49.8% of *top 5* ranked links were target links, respectively. Next, examining the *top 20* ranked links revealed that at least one target link appeared in all 400 replicates for **s1–** **s4**, and in 97 of 100 **s5** replicates (see Figure 2B). Furthermore, comparing the *top 25* link ranking to a random allocation revealed that LDWeaver performs approx. 30 times better across all scenarios (see Supplementary Figure S1).

Finally, to assess the possibility of a target link being tagged by an alternative site in genomic proximity due to LD, we calculated the genomic distance between all 10 detected sites and their closest target regions for the top 5 ranked links in each replicate (see Figure 2C). In replicates containing a target link within the top 5 ranked, this distance is 0. For others, it represents the distance to the nearest target region for the site that is in the closest genomic proximity to a target region. The analysis revealed that 94.2% of replicates contain a target link in the top 5 ranked, as indicated by a median distance of 0.

### LDWeaver detects co-evolutionary links in multiple pathways in *S. pneumoniae*.

*S. pneumoniae* is a naturally transformable nasopharyngeal commensal and respiratory pathogen. It causes a substantial global disease burden in humans, representing a major cause of pneumonia, meningitis, and otitis media. There are >100 immunologically distinct capsule types, termed serotypes, of *S. pneumoniae*. Serotype replacement after the introduction of pneumococcal conjugate vaccines (PCVs) is a serious concern, particularly given the association of many pneumococcal genotypes with multidrug resistance (33).

The LDWeaver analysis of pneumococci focussed on two populations isolated from carriage in contrasting settings: Mae La, Thailand and Massachusetts, USA. The Mae La sample comprised 2663 high quality assemblies (accessions available in Supplementary information) (32) collected from mother and infant pairs (34). The Massachusetts sample (25) comprised 616 draft genomes (accessions available in Supplementary information) of similar quality (32). Both datasets were aligned to the ATCC 700669 (accession code FM211187) reference genome (35), and after filtering out sites with minor allele frequency (MAF) < 0.01 and gap frequency > 0.15, respectively 88603 and 89386 SNPs were retained for analysis (see Supplementary Figures S2 and S3 for LDWeaver plot panels). Analysed and detected link counts are summarised in Supplementary Table S2.

Long-range interactions included strong signals of co-evolution between pbp2b and pbp2x in both populations. These genes are key in determining resistance to beta lactam antibiotics and these interactions were reported previously (12). Another interaction conserved across both populations was that between three loci encoding immunogenic surface-exposed degradative enzymes (36): the beta-galactosidase BgaA, the immunoglobulin A protease ZmpA, and PabB, encoded by a gene directly downstream of that for the ZmpA paralogue, ZmpB. These co-evolutionary signals may arise through direct interactions on the surface, or indirect effects emerging through immune selection for particular combinations of antigens (37).

The short-range interactions were more similar between the two populations. Adjacent genes functioning in the same metabolic pathway included links consistently identified between the neighbouring coding sequences cdsH (SPN23F08030) and thiI (SPN23F08040), which are both involved in thiamine biosynthesis (38). Similarly, the adjacent genes SPN23F02450 and SPN23F02460 both encode proteins predicted to function as N-acetyltransferases. Signals were also identified in both populations between the overlapping genes SPN23F08250 and SPN23F08260, encoding subunits of a transporter of unknown function (39). Hence, LDweaver can identify signals of proteins likely to be co-evolving as participants in the same metabolic pathways.

**Figure 3.**
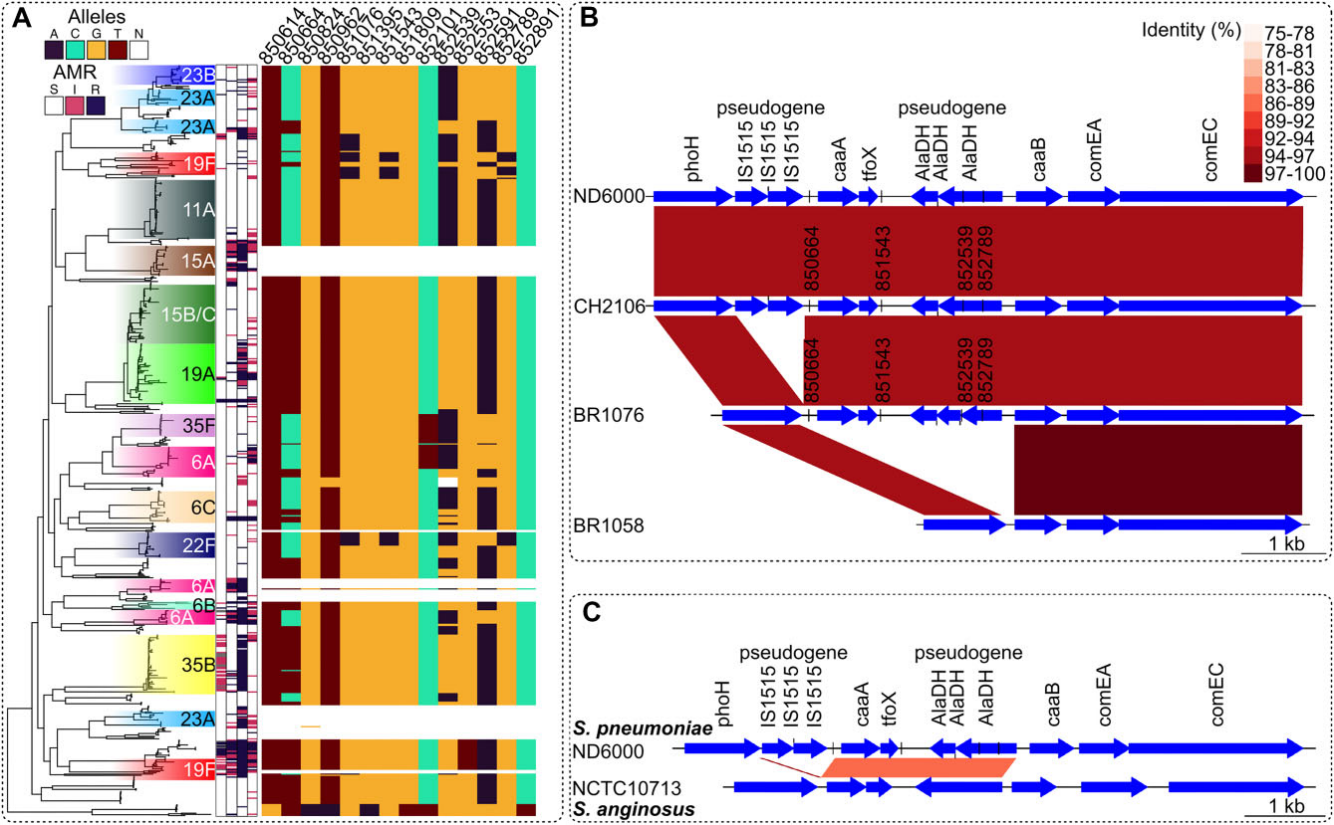
Overview of genomic variations of the flanking region of the TfoX competence regulator demonstrated using the Massachusetts S. pneumoniae dataset. (**A**) The phylogenetic tree (*n* = 616) is coloured according to the serotype shown to the right of the tree. The 4 bars immediately to the right denote the antimicrobial resistance data for ceftriaxone, erythromycin, benzyl penicillin and trimethoprim, respectively. The key above indicates the colour shadings for S – sensitive, I – intermediate and R – resistant. Allelic variation in the tfoX locus is shown by the rightmost heatmap and the key above shows the colour for each nucleotide with N indicating an ambiguous base. SNP positions above the heatmap are based on the ATCC 700669 reference. This suggests that the tfoX locus is present in most pneumococci and can be divided into three genotypes: that containing the major alleles at each polymorphic site; that containing the minor alleles at sites 850664 and 852539, and that containing the minor alleles at these two sites, as well as sites 851543 and 852789. (**B**) The alignment of representatives of the four observed genotypes in the dataset, demonstrated here using sample data from selected isolates. Colour shading indicates the identity between regions. In ND6000 (ERR129187) and CH2106 (ERR129095), the flanking region is intact with multiple insertion sequences, two functional genes, a non-coding region and an alaDH pseudogene. The locus is intact, but the insertion sequences are not observed in BR1076 (ERR129048). In contrast, the entire locus is missing in BR1058 (ERR129043). With reference to the phylogenetic tree in (A): ND6000 is a serotype 7C isolate between regions 19F and 11A, CH2106 is a 19F isolate, BR1076 is 6C isolate and BR1058 is a 7C isolate. (**C**) Shows the alignment between FM211187 and *S. anginosus* NCTC10713 genomes. For the region of interest, this is the most similar locus among streptococcal species. Despite the general divergence between *S. pneumoniae* and *S. anginosus*, the tfoX-alaDH gene pair is intact in both species with high similarity (same key as in (B) to show region identity). However, the dissimilarity between the flanking regions demonstrates the typical level of divergence between the genomes. Hence, the localized similarity indicates a possible recent introgression into the pneumococcus.

Another regulatory locus involved in short range interactions across both datasets is that encoding the competence regulator TfoX. Multiple sites were in strong LD in the subset of isolates encoding this locus, which was absent in a minority of isolates (see Figure 3A). Yet these sites are not in the gene itself, but in the flanking intergenic regions, or the adjacent alaDH pseudogene that encodes an apparently functionally unrelated alanine dehydrogenase. This suggests that these paired sites do not interact functionally. A search for the functional sequence of the alaDH gene identified intact versions in other streptococci. Further alignments indicated that the tfoX-alaDH pairing was intact in *Streptococcus anginosu*s. Hence, the variable distribution of this locus in *S. pneumoniae* is likely the consequence of one, or more, interspecies transfers through homologous recombination introducing the gene cassette into pneumococci, followed by degradation of the alaDH gene into a non-functional form (40). Hence the elevated LD identified in this case may be the consequence of a relatively recent introgression (41) from another streptococcal species, demonstrating LDWeaver can identify loci under sufficiently strong selection to drive interspecies transfers (40).

Another transporter (encoded by SPN23F03500) was linked to an adjacent pseudogene (SPN23F03510) in both populations (see Supplementary Figure S4). The undisrupted sequence of SPN23F03510 is predicted to function as a lantibiotic synthesis protein, suggesting the associated transporter is likely to be a self-immunity protein. Hence, this pairing likely represents an example of a non-producing, immune-only ‘cheater’ bacteriocin phenotype (42), common in pneumococci (43). The most variable bacteriocin-encoding locus in the pneumococcal genome is the *blp* gene cluster (44), in which multiple interactions between genes were detected. These included interactions between the genes encoding the BlpRH quorum sensing two component system, and the gene encoding the cognate peptide pheromone, BlpC (45), identified in both populations. Further links were found in the Massachusetts sample only, which likely represent relationships between the synthesized bacteriocins and corresponding immunity proteins (44).

In addition to performing biological analyses, we used the Massachusetts dataset to compare the effect of varying the CDS clustering parameter (*k*). We repeated the LDWeaver analysis using *k*= 1, 3 and 5 and fitted the decay model to the 95th percentile of the empirical distribution. Fitted parameters for each case are shown in Supplementary Table S3. Each parameter pair corresponds to the slope and intercept of the fitted model (see Materials and methods). Corresponding decay curves are shown in Supplementary Figure S5a. Next, we examined the variation between the top 250 ranked links from each analysis. Links between the same coding regions were pooled together irrespective of the ranking and the counts are shown in Supplementary Figure S5(b). Here, the qualitative difference between the choice of *k* = 1 and *k* = 3 is clear, however, choosing between *k* = 3 and *k* = 5 only results in a marginal difference.

### LDWeaver identifies patterns of co-evolution of cytolethal distending protein toxin subunits in *C. jejuni*

*C. jejuni* is a leading cause of food-borne bacterial gastroenteritis worldwide, associated with the consumption of contaminated poultry meat. It is well-adapted to colonize the gut of the majority of mammalian and avian host species and has been isolated from many environmental sources. The successful colonization of strains is often host-specific for the majority of lineages (specialist clonal complexes) and this is reflected in the population structure during phylogenetic comparison of genomes. Certain clonal complexes (CCs) are also known to be host generalists, where the same lineage is well-adapted to colonize and to survive in multiple different hosts.

The population of *C. jejuni* is structured by well-defined, genetically similar clusters of isolates (clonal complexes) which are documented to be maintained over time (26), despite the very frequent homologous recombination occurring across the known lineages (26,46). This high frequency of recombination, which is not limited to any particular region of the genome, would generally break down the LD observed in *C. jejuni* lineages and therefore, potentially disrupt co-selected/epistatic functional groups of genes throughout the genome. Despite the high recombination, the population structure has remained stable over long periods of time (26), making *C. jejuni* an excellent candidate species for the analysis of short- and long-range epistasis and co-selection links.

A collection of 1480 previously published *C. jejuni* genomes (26) consisting of 18 different human, animal, and environmental sources and 37 CCs were selected for LDWeaver analysis (accessions available in Supplementary information). These were aligned against the NCTC 11168 reference genome (47) using snippy. After removing sites with MAF < 0.01 and gap frequency > 0.15, 102591 SNPs were retained for LDWeaver analysis (see Supplementary Figure S6 for the LDWeaver plot panel).

Analysis of short-range interactions identified strong signals of co-evolution between genes with functions mostly related to virulence such as: amino acid ABC transporters, flagellar biosynthesis, periplasmic and outer membrane proteins, antibiotic efflux genes, among others. One of the most promising findings was multiple highly significant links located within the cytolethal distending toxin (CDT) genomic region. The CDT is a protein toxin composed of three subunits: CdtA, CdtB and CdtC encoded by a cdtABC operon, and is considered one of the most important virulence factors for Campylobacter pathogenesis. CDT acts to halt host cell division by cell cycle arrest at the G_2_ stage occurring before mitosis (48). CdtA and CdtC are anchored into the membrane and act to deliver CdtB to the host cell which arrests the cell cycle. CdtB has the toxin activity but is reliant on CdtA and CdtC for its binding and delivery to the host cell (49).

**Figure 4.**
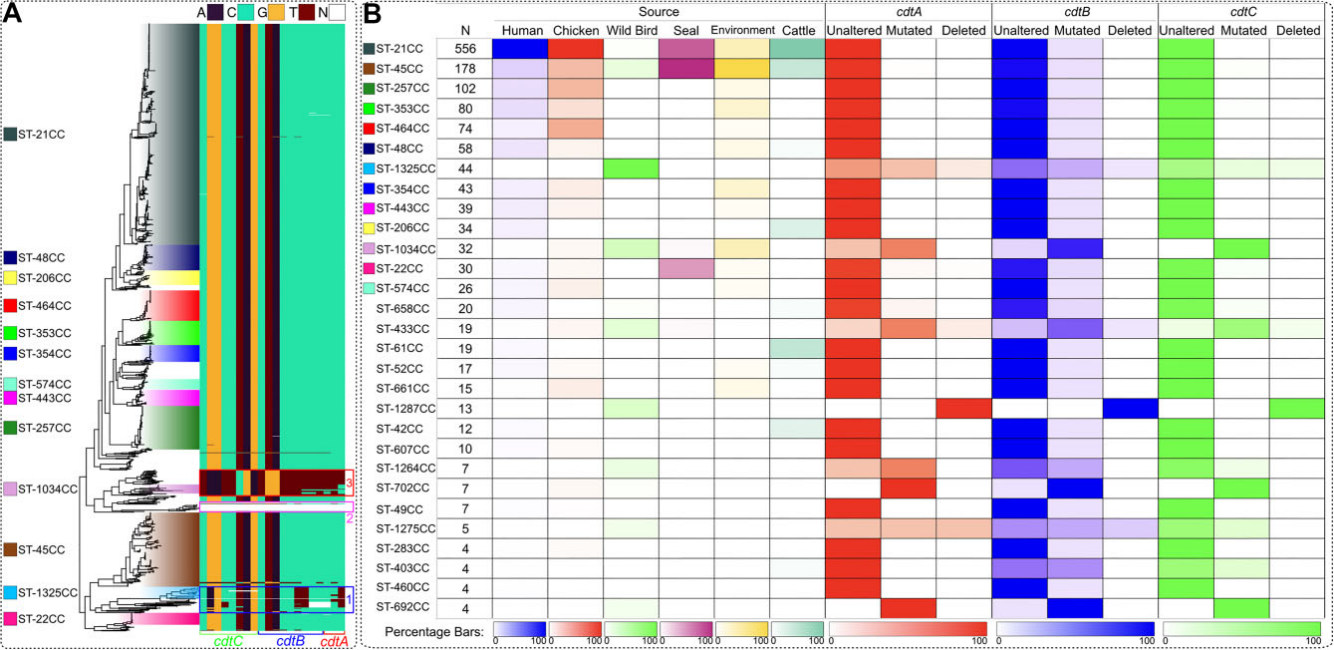
Overview of allelic variation of CDT subunits in the *C. jejuni* dataset. (**A**) The phylogenetic tree (*n* = 1480) is coloured according to the clonal complex and the key to the left of the tree is labelled according to the corresponding topology of each clonal complex. Allelic variation of the three CDT subunits (CdtA, CdtB and CdtC (*x axis*)) encoded by the *cdtABC* operon across the tree is represented by the heatmap to the right of the tree. (**B**) The association of a particular clonal complex with a source is highlighted by the first panel. The darker the shading (explained by percentage keys at the bottom of the panel), the higher the percentage of isolates from each source belonging to the particular clonal complex (rows). The rows are ordered by abundance (column *N*) in the *C. jejuni* dataset. Three panels to the right represent the allelic variation within CdtA (red), CdtB (blue) and CdtC (green) subunits. The shading represents the percentage of isolates associated with a particular clonal complex/source with either: 1) the unaltered allele; 2) a nucleotide change (mutated); or, 3) a deletion of the CDT subunit. Shaded keys at the bottom of the three panels represent the percentage of isolates.

Our results showed that the three subunits were well conserved throughout the dataset, except for three distinct clusters of isolates identified in wild birds (see Figure 4). There was high variability of the presence/absence and allelic variation of the three CDT subunits across these three clusters of isolates. For example, all three subunits were absent from the wild bird-associated ST-1287 CC. Another wild bird-associated clade, ST-1034 CC (mixed) consisted of synonymous/non-synonymous nucleotide changes in all subunits compared with other sources. Finally, isolates belonging to the third wild bird-associated clade, ST-1325 CC consisted of a mix of both the same synonymous/non-synonymous nucleotide changes as seen in ST-1034 CC, some of the isolates in this cluster also exhibited the same variation as observed in the other sources and CCs within the dataset, while some isolates had an absence of various CDT subunits (see Figure 4). A recent study comparing the gene sequences of the *cdtABC* operon of wild bird, broiler chicken and human sources (50) confirms the LDWeaver-generated hypothesis of significant co-selection occurring within this operon. The study identified high variability of *cdtABC* alleles in wild birds with several alleles producing no functional CDT. Sequence conservation outside of wild bird sources, such as broiler chickens and humans, was also observed, suggesting that the variation of the *cdtABC* operon may play a role in the host range of *Campylobacter* (50).

Potential co-selection was also identified in the gene cluster containing genes *metE* and *metF* (see Figure 1). These genes are located on an operon involved in methionine synthesis which has been proven to have a vital role in the colonisation of *C. jejuni* to the gastrointestinal tract of different hosts (Kelley *et al.*, 2021). Similar patterns of the presence/absence and mutated versions of this locus were observed within the same wild bird clusters as the *cdtABC* operon (Figures 1H and 3) while also remaining relatively conserved in the remaining sources and CCs.

In addition to performing biological analyses, in *C. jejuni*, we also explored the impact of the choice of background LD modelling (LDWeaver approximate vs. using a neutral simulation) on short-range outlier ranking (Supplementary Figure S7). Link ranking was robust to the modelling choice for generally high LD links (MI > 0.5) (Supplementary Figure S7a), however, the LDWeaver model allocates systematically higher ranks to links that are further apart (Supplementary Figure S7b). Given the primary goal of short-range epistasis analysis is to accurately rank high LD outliers in genomic proximity, these findings indicate that the choice of background LD modelling has a minimal impact. Furthermore, both approaches on average allocate the same score to links between same site pairs (Supplementary Figure S7c).

### LDWeaver recapitulates co-evolving links involved in clade evolution and success of the *E. coli* pandemic clone ST131

To investigate epistasis and co-selection in *E. coli*, we considered a dataset consisting of 2156 ST131 genome assemblies (accessions available in Supplementary information) aligned against the EC958 reference genome (51) using Snippy. Loci with MAF ≥0.01 and gap frequency <0.15 were included, leading to 44 092 SNPs (see Supplementary Figure S8 for the LDWeaver plot panel). It is noted that the *E. coli* dataset had the highest amount of LD among all analysed datasets, and the asymptote of Supplementary Figure S8b cluster 1 has the largest intercept among all LDWeaver panel plots.

The *E. coli* ST131 lineage belongs to the phylogroup B2 that emerged globally around 20 years ago and is associated with urinary tract (UTI) and bloodstream infections (BSI) (52–54). Large epidemiological studies have identified major differences in the virulence and antibiotic resistance between the three main clades of ST131 (A, B and C) (55). Clade C, which is associated with fluoroquinolone resistance arising from mutations in *gyrA* and *parC* has further been split into two sub-lineages (C1 and C2) with a distinct pattern of mobile genetic elements (MGEs) and associated antimicrobial resistance (AMR) genes (56,57).

The long-range loci pairs in *E. coli* ST131 corresponded to clade C specific SNPs differentiating sub-lineages C1 and C2 (58). These included links between sites in *sbmA* (EC958_0513), a transporter involved in the internalisation of peptide antibiotics into the cytoplasm (59) and identified as a virulence factor in avian extraintestinal *E. coli* (APEC) (60), and sites in (i) *nikA* (EC958_3870), a periplasmic protein from the ATP-binding cassette type nickel transport system acting as the initial receptor of nickel (61), (ii) *acrF* (EC958_4822) encoding an efflux pump with homology to the major pump AcrB (62), (iii) *lepA* (EC958_2875) encoding a conserved GTPase with a role in the initiation phase of translation (63) and (iv) *iscS* (EC958_2841), encoding a cysteine desulfurase implicated in the activity of a number of Fe-S proteins (64). These clade-specific SNP pairs are spread across the *E. coli* chromosome, separated by at least 1 Mbp, and may have contributed to the recent expansion of the *E. coli* sub-lineage C2.

**Figure 5.**
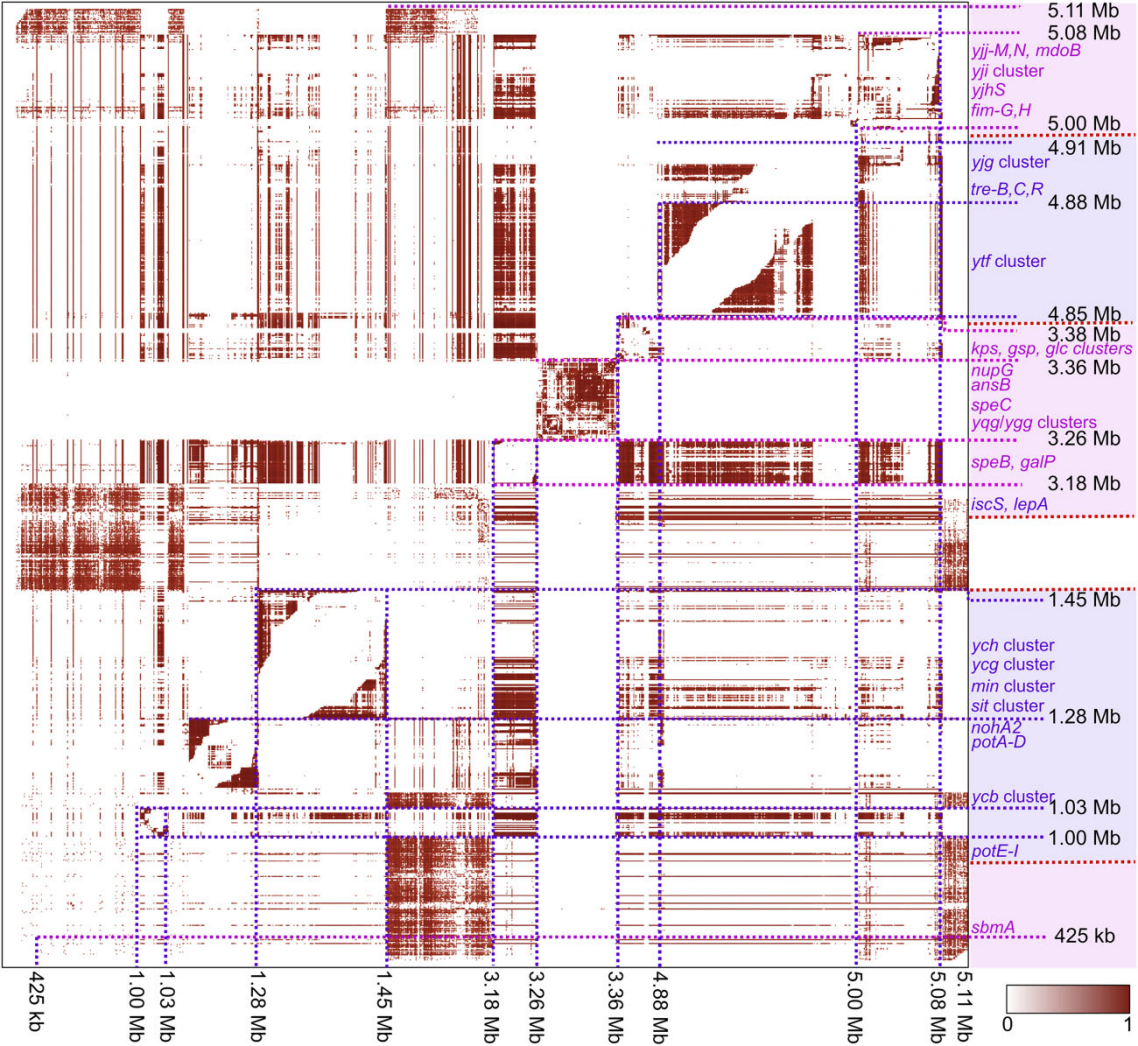
Genome-wide distribution of excess LD in the *E. coli* ST131 dataset (*n* = 2156) measured using LDWeaver. Approximate genomic positions are marked to the right and bottom of the LD map as per the EC958 reference genome. Brown shading indicates the amount of LD between sites (see key at bottom right for MI values scaled between 0 and 1). Entire genomic regions without excess LD are dropped from the figure to enhance the visibility of variation within high LD regions and each new region is coloured in an alternating shade of blue and purple in the right panel. Additionally, the right panel shows genomic regions comprising short-range excess-LD links.

The genome wide distribution of LD estimated from LDWeaver revealed multiple interesting patterns (see Figure 5). For example, the short-range co-evolving SNP pairs highly ranked by LDWeaver correspond to regions involved in the synthesis of the *E. coli* capsule polysaccharide (*kpsM* EC958_3343, *kpsC* EC958_3337, *kpsS* EC958_3338*)* and type II secretion system located downstream in the chromosome (*gspL* EC958_3345, *gspM* EC958_3344). The observed tight linkage in the *E. coli* capsular region might be critical for having a functional system since the capsule plays an important role as a major virulence factor contributing to

the colonization of different eukaryotic host niches, reducing the efficacy of the immune system by complement inactivation and shaping the horizontal gene transfer mediated by MGEs (65–67). These results indicate that the variation within the capsule region is also

linked to SNPs present in the conserved type II secretion system. Variation in these regions could thus alter the capsule expression in *E. coli*, contributing to a non-capsulated state that allows the introduction of a new pool of MGEs (67).

### LDWeaver detects novel interactions between sites associated with virulence in *E. faecalis*.

*E. faecalis* represents a classical generalist microorganism, with little phylogenetic divergence and limited host specialization over the population (68). Few hospital-associated *E. faecalis* clusters have been identified, with some overrepresentation of virulence factors and AMR genes (28,69). However, these lineages as well as the traits potentially underlying their success predate the modern hospital settings (28,70). The *E. faecalis* dataset analysed using LDWeaver comprised 2027 isolates (accessions available in Supplementary information) aligned against the V583 reference genome (71) using Snippy. After filtering out sites with MAF < 0.01 and gap frequency > 0.15, we analysed 85982 SNPs (see Supplementary Figure S9 for the LDWeaver plot panel).

Inspecting the short-range links within the *E. faecalis* population revealed multiple top-ranking links in known enterococcal virulence genes. Particularly, the elr operon was represented in several links (see Supplementary Figure S10). The genes in the *elr* operon code for putative surface proteins and their overexpression have been associated with increased virulence and ability to evade host immune defence by resistance to phagocytosis (72). Three *elr* genes, *elrB, elrC* and *elrR*, were linked to each other, and the positive regulator *elrE* (73) was also linked to an ATP-binding cassette transporter.

Another cluster of links involved *ace*, a widespread virulence gene in *E. faecalis*, coding for adherence factor (74,75). In addition to a gene coding for a hypothetical protein, it was linked to a bacteriocin-encoding *entV*. This bacteriocin has been shown to act against fungal *Candida albicans* co-infection by the inhibition of biofilm formation (76,77). Intriguingly, the absence of ace has also been shown to result in reduced biofilm formation *in vivo*, but despite both *ace* and *entV* being partly involved in biofilm-associated enterococcal infections (78), we are not aware of any report of a direct link between the functions of the two. These findings demonstrate the ability of LDWeaver to highlight both known and putative functional links between enterococcal genes. Supplementary Figure S10 further illustrates that the minor allele haplotypes at the candidate sites under co-selection are not enriched in hospital-associated multi-drug resistant lineages. Given that the ages of these lineages have been estimated as 50–150 years (28), and that they all share the major alleles at these variable sites, the co-selective pressure may have acted more recently in some other ecological setting outside hospitals.

## Discussion

Bacterial population genomics research is rapidly moving towards an era where hundreds of thousands of whole-genome sequences will be available for many species. These data represent an unprecedented opportunity to seek signals of selection in natural populations and to unravel genomic clues for adaptation under changing ecology, which can contribute towards improved understanding about evolution, dissemination and maintenance of antibiotic resistance and virulence traits. Existing GWES methodology has already enabled discoveries of co-selection affecting polymorphisms across distant genomic sites in a variety of human pathogens (13–15). By extending GWES to joint screening of polymorphisms in close proximity, we increase the potential of data-driven molecular discovery for bacterial populations, which are particularly challenging for GWAS due to the difficulty of large-scale measurement of traits.

Applying our methodology across a diverse spectrum of bacterial species: *S. pneumoniae*, *C. jejuni*, *E. coli* and *E. faecalis*, has revealed fresh insights into genomic interactions between proximate variants. Notably, our results were obtained without use of phenotype data and would not be detected via conventional genome-wide association (GWAS) methods as they would typically be discarded due to the influence of short-range linkage disequilibrium. Our results included findings associated with host range, antibiotic resistance, virulence, and immune evasion. Furthermore, our top results often represented links from genomic islands: *tfoX* in *S. pneumoniae*, *cdtABC* in *C. jejuni*, capsular locus in *E. coli* and *elr* genes in *E. faecalis*, all of which are self-contained units that may evolve differently to the rest of the chromosome due to reduced functional integration. While these novel potential interactions require experimental validation, our approach drastically reduces the number of pairwise relationships that need to be considered. Recent advances in computational protein structure prediction could further reduce the need for time-consuming wet-lab experiments by rapidly considering the impacts of the identified intragenic interactions on the resulting protein structure.

A potential target for further development of genome-wide short-range LD analysis is to consider genomic variation beyond reference-based core genome alignments. With the increasing availability of long-read based assemblies of chromosomes and plasmids, it would be attractive to consider detection of co-selection both within plasmids and between plasmid and host chromosome polymorphisms, to potentially uncover either compensatory evolution or pre-adaptation to stable carriage of particular plasmids (79).

Furthermore, while wet-lab-based validation will remain the gold standard to verify combined effects of mutations, future developments should incorporate information beyond the DNA sequence, such as gene expression levels, protein structure alterations and epigenetic variations, which has the potential to greatly contribute towards improved detection. Using only DNA sequence information about LD is limiting because, in addition to selection acting on combined sets of mutations, LD signals can reflect evolutionary factors such as population expansion, elevated mutation rates, and hitchhiking.

Given its existing and future potential for enabling molecular discoveries, we anticipate that GWES will continue to attract a wide interest from both methodological and applied perspectives.

## Supplementary Material

lqae061_Supplemental_Files

## Data Availability

LDWeaver v.1.5 is available as an R package under a GNU General Public License (Version 3) on GitHub (https://github.com/Sudaraka88/LDWeaver) and the source code is available on Zenodo (https://zenodo.org/records/10016711). Genomic data accessions used in this analysis are available via figshare (https://doi.org/10.6084/m9.figshare.24079491). An interactive phylogenetic tree (Figure 3A) clearly marking the detected isolates can be accessed on microreact (https://microreact.org/project/s94ZeKRZUSkz7JZrkwsuFY-spnmschtree).
