# Supplementary material for: Detecting co-selection through excess linkage disequilibrium in bacterial genomes
Source: NAR Genom Bioinform. 2024 Jun 6;6(2):lqae061. doi: 10.1093/nargab/lqae061 (PMC11155488; doi:10.1093/nargab/lqae061)
Supplement: lqae061_Supplemental_Files [file lqae061_supplemental_files.zip › NARGAB-2024-038.R1Supplementary.pdf]

Supplementary Information

Scenario	Detectable target link percentage: mean (SD)	Detected target link percentage in *top 25 ranked:* mean (SD)	Detected non-target links percentage in *top 25 ranked:* mean (SD)
**s1**	2.42 (0.58)	64.2 (16.3)	35.8 (16.3)
**s2**	1.98 (0.51)	55.8 (15.4)	44.2 (15.4)
**s3**	1.77 (0.54)	53.0 (15.6)	47.0 (15.6)
**s4**	1.57 (0.56)	49.0 (16.4)	51.0 (16.4)
**s5**	1.31 (0.55)	43.8 (20.1)	56.2 (20.1)

Supplementary Table. 1 Performance benchmarks for simulation scenarios. Number of assessed links per replicate in thousands: median 258.84, mean 284.83, sd 135.29, min 65,341, max 928.20. Across all simulations, 53.1% of *top 25 ranked* links are target links. Even in scenario s5, which has approximately half the number of detectable target links compared to s1, LDWeaver manages to fill 43.8% of the *top 25 ranked* links with target links - a random allocation would have only filled 1.31% on average.

Dataset	Seqs	SNPs	Total pairwise links	Outlier Links (short range)	Short range - Direct	Short range - nSyn	Outlier Links (long range)	Long range - Direct	Long range - nSyn
S. pneumoniae (Maela)	2,663	88,603	3,925,201,503	348,899	14,433	7,115	21,299	3,028	1,282
S. pneumoniae (Msch)	616	89,386	3,994,883,805	152,116	18,224	8,729	42,599	11,530	6,741
C. jejuni	1,480	102,591	5,262,405,345	495,196	42,062	18,890	27,819	3,621	1,575
E. coli	2,156	44,092	972,030,186	25,054	833	288	65,942	24,331	5,774
E faecalis	2,027	85,982	3,696,409,171	246,863	11,094	5,053	240	48	18

Supplementary Table. 2 Summary of datasets and the number of links detected. LDWeaver reduces the scope from having to scan potentially **billions** of genome-wide pairwise links to several thousands (or hundreds in some cases). In many cases, these thousands of links are from the same two genomic regions due to the high LD between them. Collapsing and visualising these regions can be performed using the LDWeaver network plot (see Fig. 1g), which will limit the search even further. Additionally, biological knowledge can be used to narrow the search radius even further - this is the approach followed when analysing the biological results presented in this manuscript.

k	Cluster 1	Cluster 2	Cluster 3	Cluster 4	Cluster 5
1	-0.252, -0.263				
3	-0.226, -0.490	-0.249, -0.264	-0.260, -0.346		
5	-0.224, -0.539	-0.230, -0.379	-0.272, -0.140	-0.248, -0.495	-0.278, -0.197

Supplementary Table. 3 LDWeaver model fit coefficients for three clustering configurations. The Massachusetts *S. pneumoniae* dataset was analysed thrice by varying the number of CDS clusters (k). For each case (k = 1,3,5), this table shows the slope and intercept parameters by fitting the following model: $\log log\left(q_{95}\right) \sim \log log\,(bp\_sep)$. Here, q_{95} is the 95th percentile of the empirical distribution and *bp_sep* is the base pair separation (see methods). Supplementary Fig. 5(a) shows the corresponding decay curves.

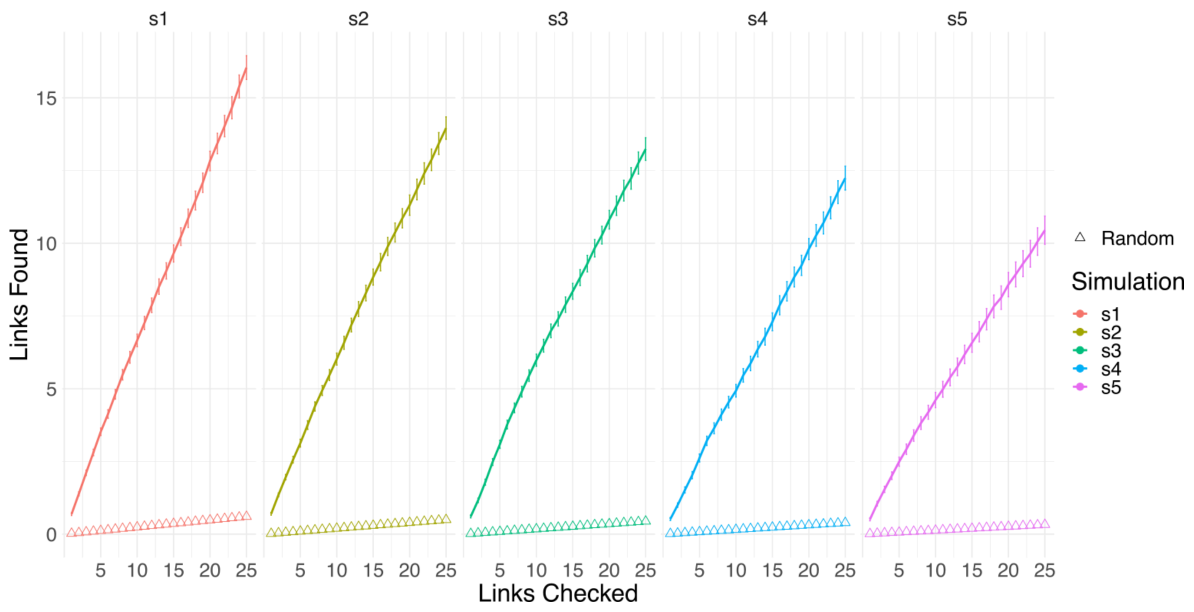

Supplementary Figure 1. Comparison of LDWeaver links ranking with a random approach. For each simulation scenario (s1-s5), the number of links detected (*y*-axis) is shown against the number of links examined (*x*-axis). Continuous coloured lines (above) show the LDWeaver performance for each scenario. Error bars on the show the standard error in the number of detected target links between replicates. Triangles (below) show the average number of detectable links by a random allocation. Compared to the random allocation, LDWeaver performs approx. 30 times better within the top 25 link rankings.

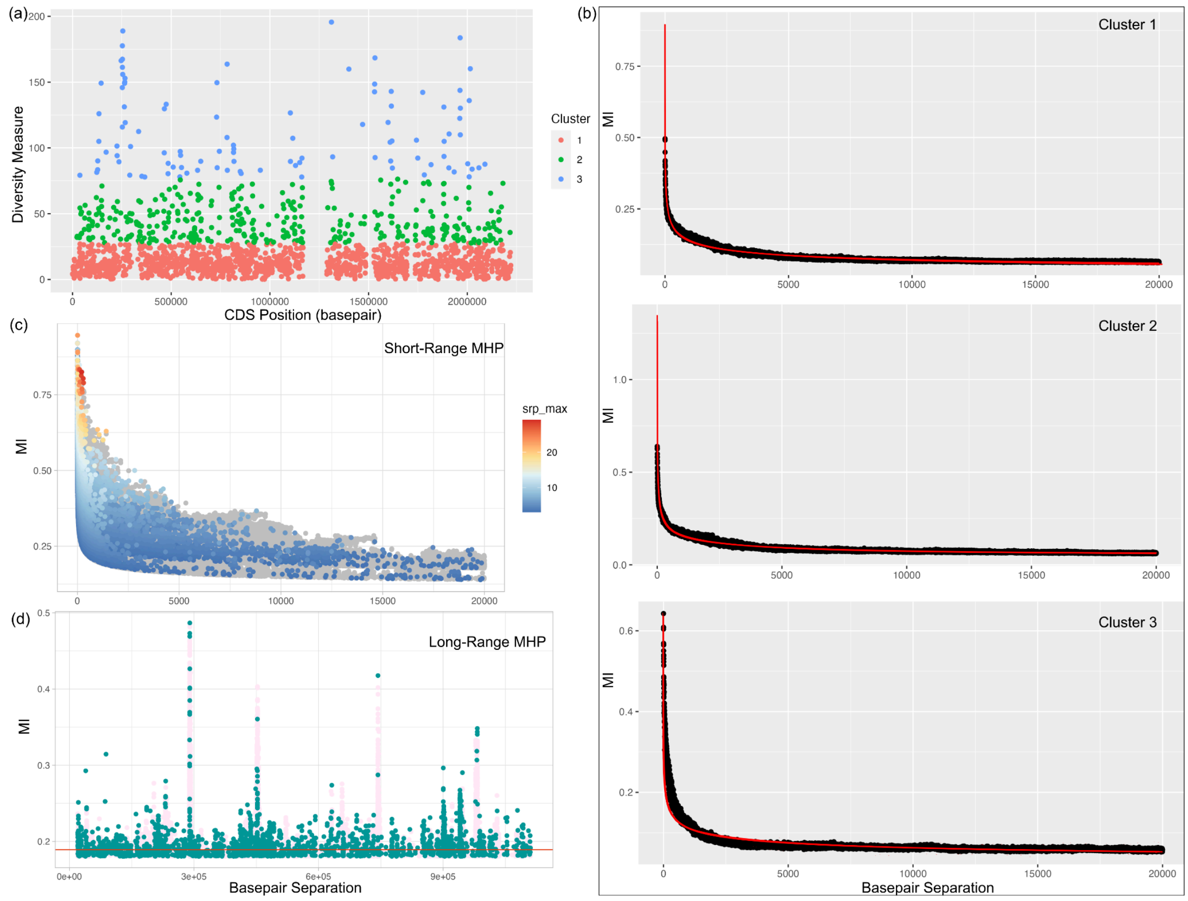

Supplementary Figure 2. Panel of LDWeaver plot for the *Streptococcus pneumoniae* Maela dataset aligned using the ATCC700669 reference genome. (a) Diversity measure of each CDS in the reference genome annotation. K-means clustering was performed to allocate each CDS to one of three clusters (colours) based on the diversity. (b) For each cluster, the panel shows the LD-decay of the empirical 95th percentile MI value at each bp-sep (black dots) and the fitted values $\hat{q}_{95}$ (red line). (c) In the short-range Manhattan plot, the *x*-axis shows the bp-sep and the *y*-axis shows the MI value. Each coloured point corresponds to an outlier link in the dataset. Links are coloured according to the srp value computed using the background-LD distributions in (b). When a link stems from two clusters, srp is computed for both backgrounds and the maximum value is used. Points in ash colour are ARACNE indirect links. (d) The long-range Manhattan plot conveys similar information to (c) and the red horizontal line shows the Tukey outlier cut-off.

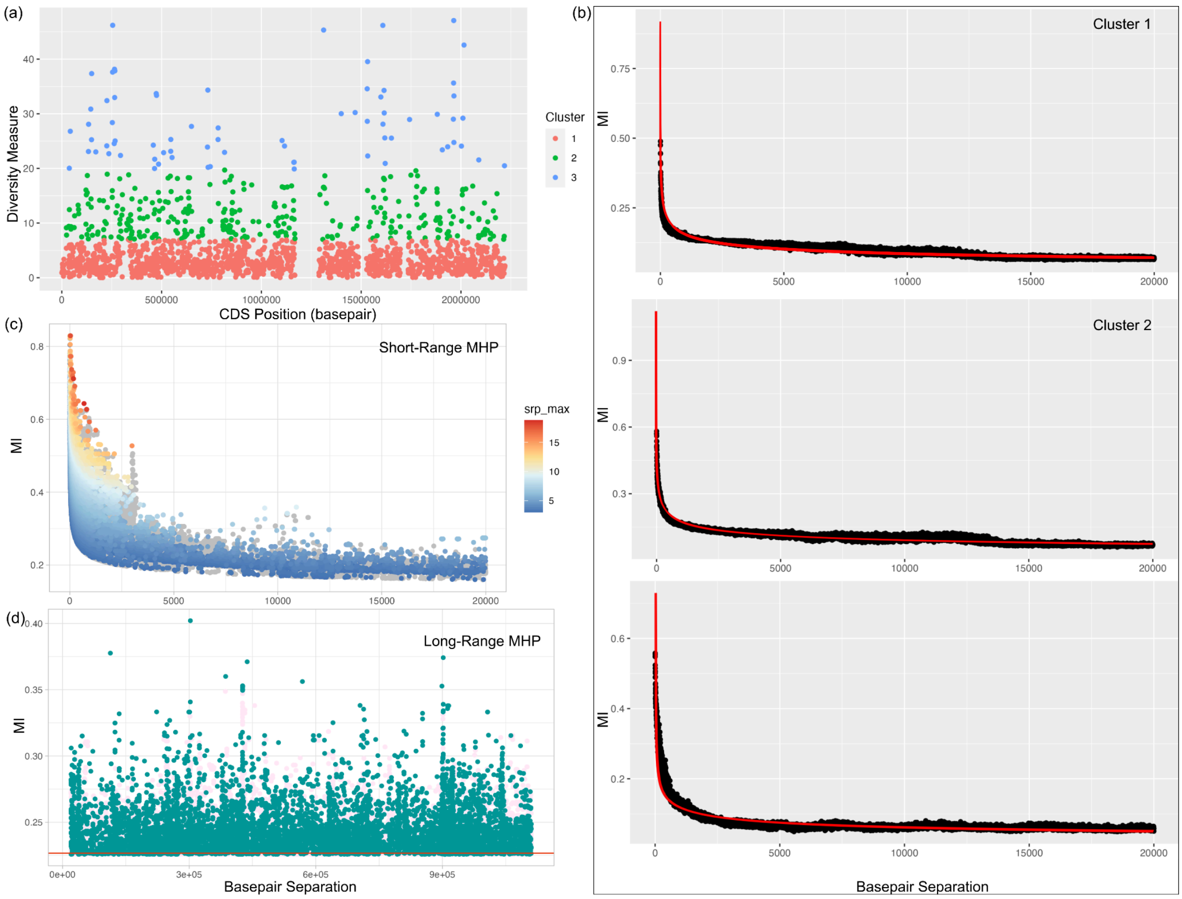

Supplementary Figure 3. Panel of LDWeaver plot for the *Streptococcus pneumoniae* Massachusetts dataset aligned using the ATCC700669 reference genome. See Supplementary Figure 2 caption for figure details.

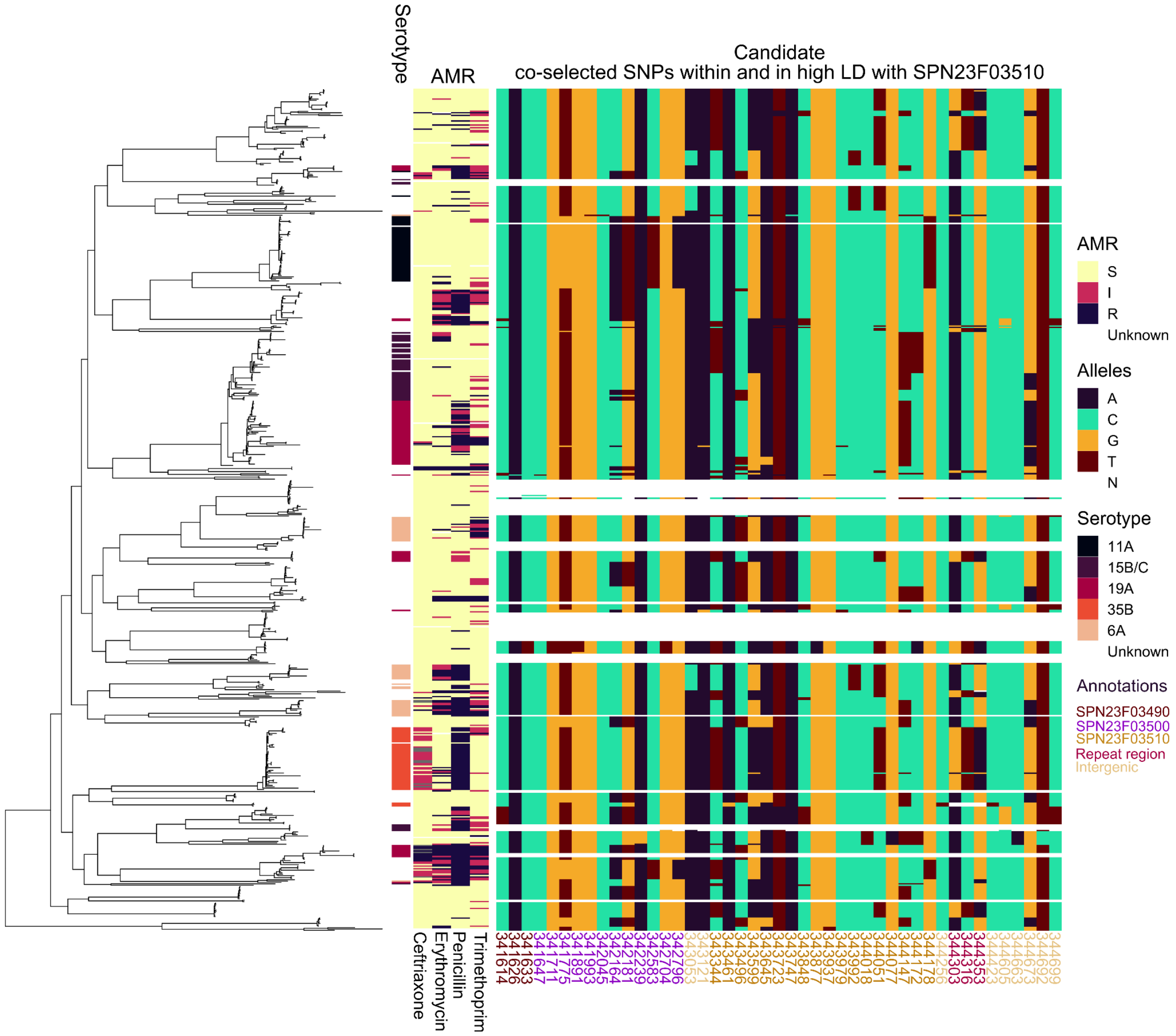

Supplementary Figure 4. Overview of the allelic variation within SPN23F03490 - SPN23F03510 region in the Massachusetts *S. pneumoniae* dataset. (a) The phylogenetic tree (n = 616) was estimated using FastTree 2 [102] and the 5 bars immediately to the right denote the serotype, and antimicrobial resistance data for ceftriaxone, erythromycin, benzyl penicillin and trimethoprim, respectively (keys are shown on the right and the AMR colour shadings indicate S - sensitive, I - intermediate and R - resistant). Allelic variation is shown by the heatmap to the right and the corresponding key shows the colours for nucleotides with N indicating gap. SNP positions below the heatmap are based on the FM211187 reference and are colour coded to indicate the annotation (key on the right).

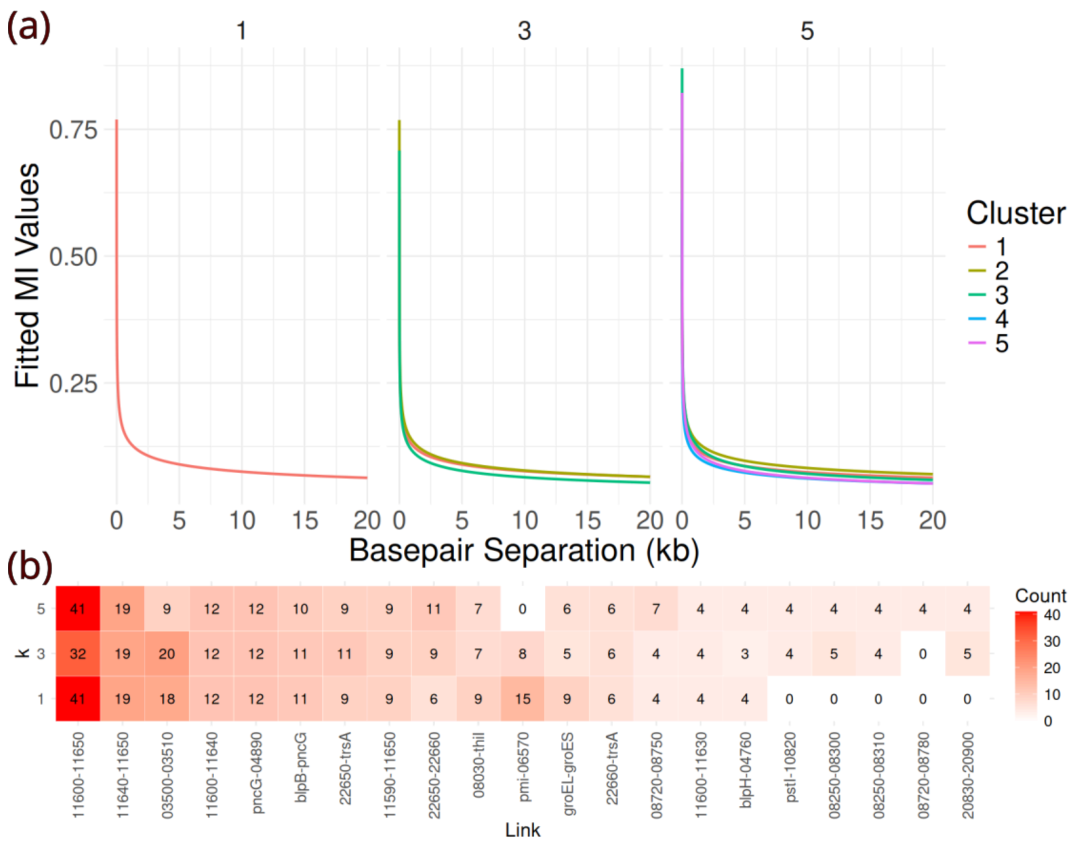

Supplementary Figure 5. Fitted decay curves for the Massachusetts dataset for three clustering configurations and the effect on link ranking. (a) shows the decay curves plotted using model fit parameters in Supplementary Table 3. Here, the three plots (left-right) show the modelling scenarios *k* = 1, 3 and 5 and each cluster is coloured according to the legend on right. (b) The number of links (x-axis) ranked in the top 250 detected for each option of *k* (y-axis). Here, links between the same two coding regions were pooled together irrespective of the ranking and to maintain readability, site pairs with <4 links were discarded. Here, the choice of *k* = 3 and *k* = 5 does not make a considerable difference but *k* = 1 fails to capture a substantial degree of variation.

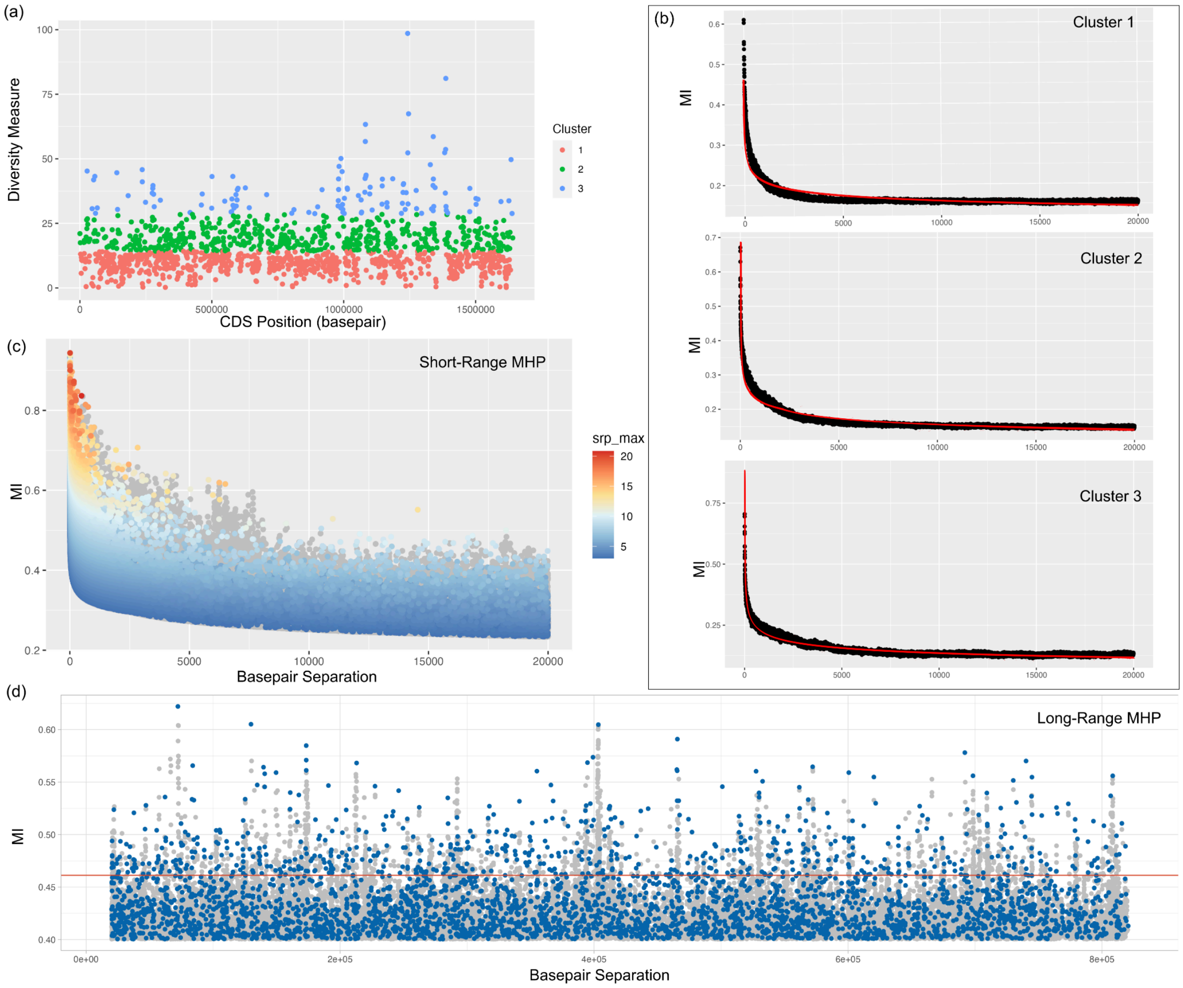

Supplementary Figure 6. Panel of LDWeaver plot for the Campylobacter jejuni dataset aligned using the NCTC 11168 reference genome. See Supplementary Figure 2 caption for figure details.

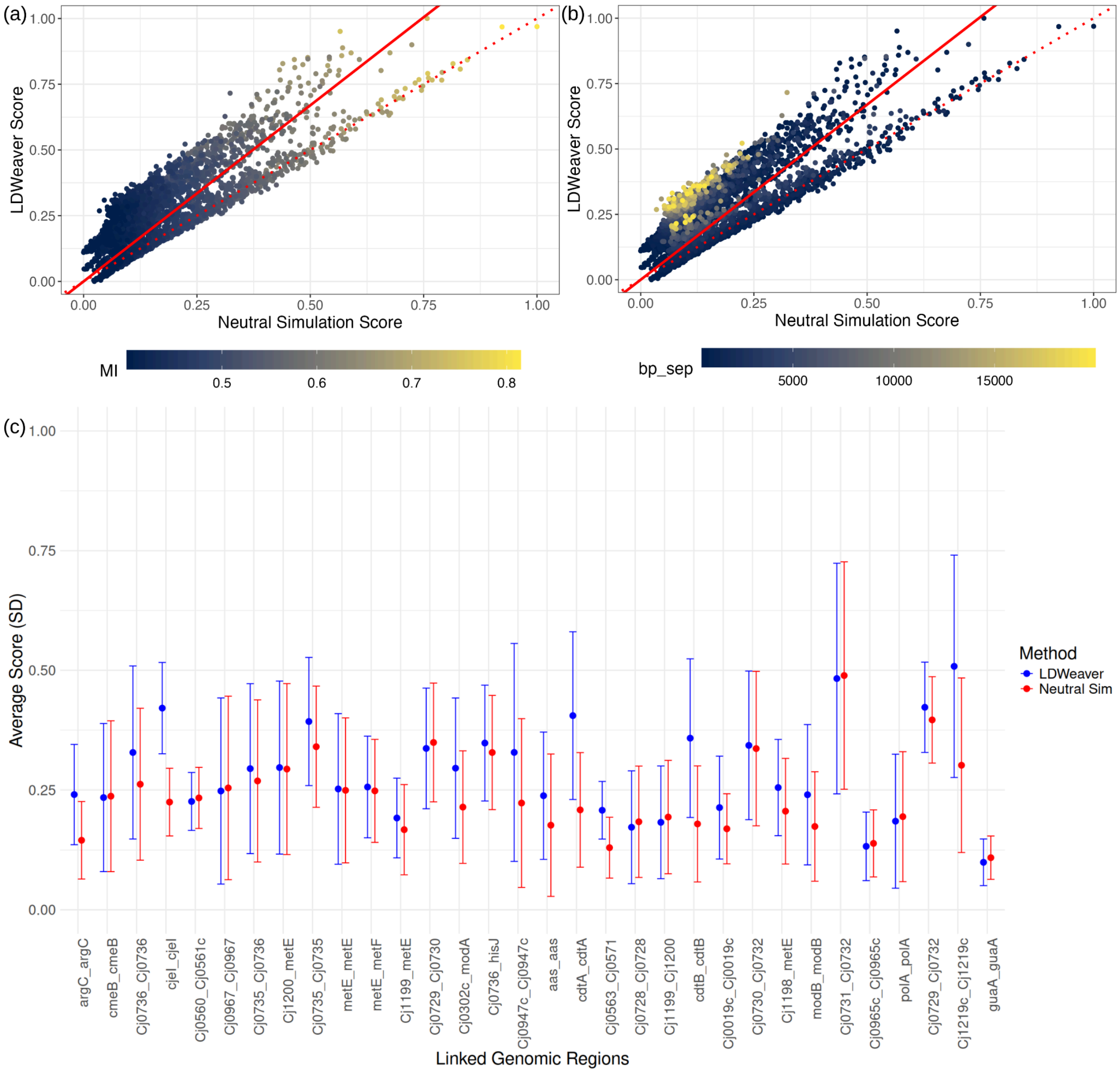

Supplementary Figure 7. Comparing the link ranking effect on *C. jejuni* short-range outliers based on the choice of background LD model fit. In both (a) and (b), each point refers to a detected outlier link. Both *x*-axis and *y-axis* show normalised link ranks, where the ranks are normalised by dividing by the maximum rank. Here, *y*-axis and x-axis show the outcome from two background LD modelling scenarios; *y*-axis: LDWeaver approximate fit, *x*-axis neutral model fit. The red lines show x=y (dotted) and the best fit (solid). Colour shading indicates the (a) LD (measured in MI) and (b) genomic distance (measured in base pair separation). Generally, links in high LD (MI>0.5) are ranked

similarly using both approaches, but links that are further apart are given higher ranking by LDWeaver compared to the neutral modelling approach. The fitted linear regression slope = 1.34 with SE = 0.007 and $p < 0.001$. (c) Genomic site pairs (x-axis) with >15 outlier links are shown, arranged from left to right in decreasing order of the number of links (y-axis). The y-axis uses the same scores from (a) and (b) and the error bars show the SD. In a majority of site pairs, there is no significant difference in average ranking between the two methods.

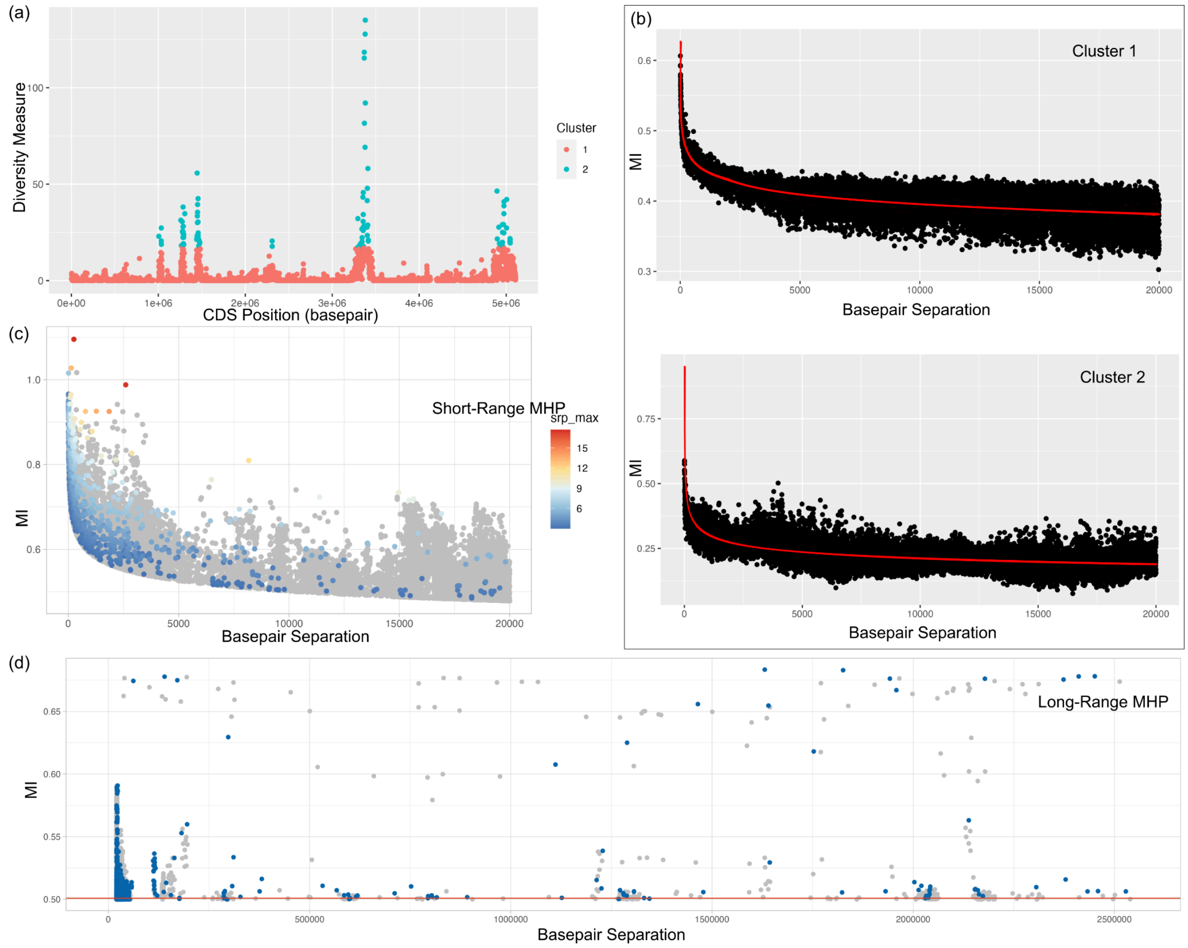

Supplementary Figure 8. Panel of LDWeaver plot for the *Escherichia coli* dataset aligned using the EC 958 reference genome. See Supplementary Figure 2 caption for figure details.

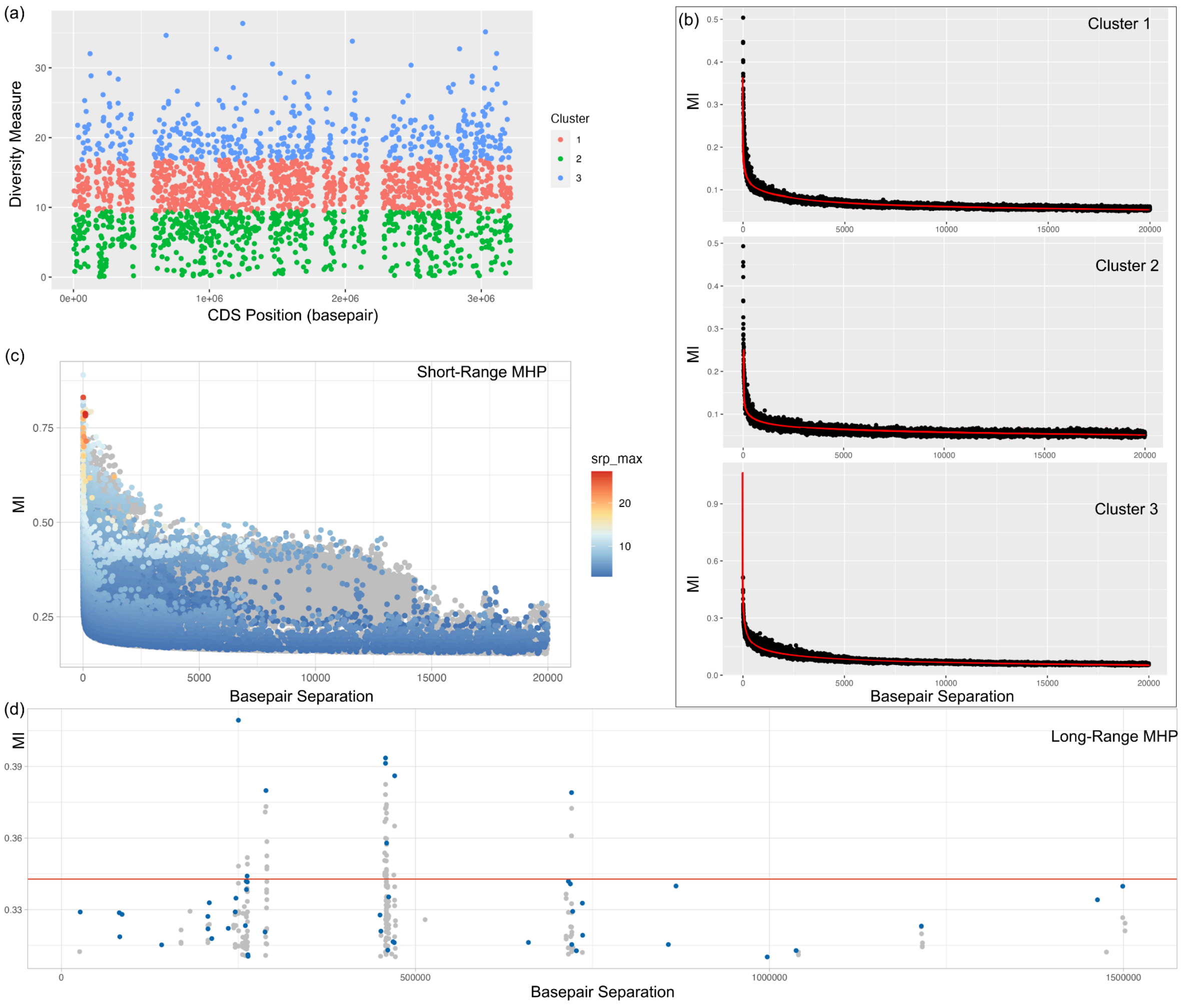

Supplementary Figure 9. Panel of LDWeaver plot for the *Enterococcus faecalis* dataset aligned using the V583 reference genome. See Supplementary Figure 2 caption for figure details.

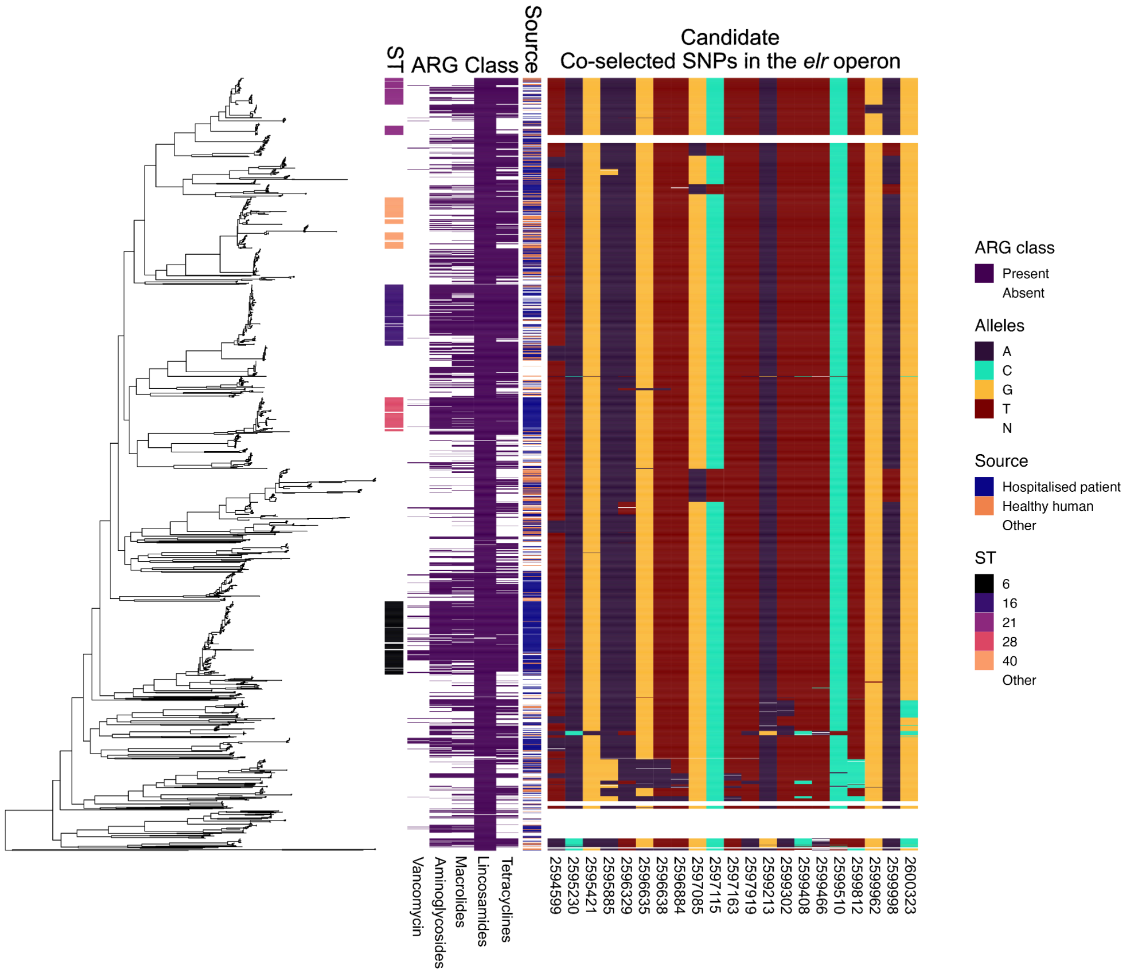

Supplementary Figure 10. Approximate maximum likelihood phylogeny for the core-genome alignment, estimated using FastTree 2 [102]**, aligned to panels of metadata and a subset of alleles in the *elr* operon.** From left to right, panels show, from left to right ST - sequence type, ARG class - Antimicrobial Resistance Genes (see below for each ARG), Source of isolate and the Allele distribution for 21 SNPs (see below for genomic position as per the V583 NC_004668.1 genome). All SNPs shown in the panel were detected as short-range top ranked links by LDWeaver.
